# The viscoelastic paradox in a nonlinear Kelvin–Voigt type model of dynamic fracture

**DOI:** 10.1007/s00028-024-00989-0

**Published:** 2024-07-08

**Authors:** Maicol Caponi, Alessandro Carbotti, Francesco Sapio

**Affiliations:** 1 Dipartimento di Matematica e Applicazioni “R. Caccioppoli”, Università degli Studi di Napoli “Federico II”, Via Cintia, Monte S. Angelo, 80126 Naples, Italy; 2 Dipartimento di Matematica e Fisica “E. De Giorgi”, Università del Salento, Via Per Arnesano, 73100 Lecce, Italy; 3 Wolfgang Pauli Institute, Oskar-Morgenstern-Platz 1, 1090 Vienna, Austria

**Keywords:** Dynamic fracture, Cracking domains, Elastodynamics, Nonlinear viscoelasticity, Monotone operators, Energy-dissipation balance, Viscoelastic paradox, 35L53, 35A01, 35Q74, 47H05, 74D10, 74R10

## Abstract

In this paper, we consider a dynamic model of fracture for viscoelastic materials, in which the constitutive relation, involving the Cauchy stress and the strain tensors, is given in an implicit nonlinear form. We prove the existence of a solution to the associated viscoelastic dynamic system on a prescribed time-dependent cracked domain via a discretization-in-time argument. Moreover, we show that such a solution satisfies an energy-dissipation balance in which the energy used to increase the crack does not appear. As a consequence, in analogy to the linear case this nonlinear model exhibits the so-called *viscoelastic paradox*.

## Introduction

In the derivation of a mathematical model for dynamic crack propagation, the two fundamental facts that must be taken into account are the laws of elastodynamics and the (dynamic) Griffith criterion. The first one states that the displacement of the deformation must solve the elastodynamics system away from the crack, while the second one dictates how the crack grows in time. More precisely, the Griffith criterion (see [16, 18]), originally formulated in the quasi-static setting, explains that there is a balance between the mechanical energy dissipated during the evolution and the energy used to increase the crack, which is supposed to be proportional to the area increment of the crack itself.

The first step to address the study of a model of dynamic fracture is to find the solution to the elastodynamics system when the evolution of the crack is prescribed. From the mathematical point of view, this leads to the study of the following system in a time-dependent domain:

1.1 $$\begin{aligned} \ddot{u}(t)-\mathop {\textrm{div}}\nolimits (\sigma (t))=f(t)\quad \text {in}\ \Omega \setminus \Gamma _t, t\in [0,T], \end{aligned}$$

with some prescribed boundary and initial conditions. In the above formulation, $\Omega \subset {\mathbb {R}}^d$ is an open bounded set with Lipschitz boundary which represents the reference configuration of the material, $\Gamma _t\subset {{\overline{\Omega }}}$ is a $(d-1)$-dimensional closed set that models the crack at time *t*, $u(t):\Omega \setminus \Gamma _t\rightarrow {\mathbb {R}}^d$ is the displacement of the deformation, $\sigma (t)$ is the Cauchy stress tensor, and *f*(*t*) is a forcing term. Once found the displacement *u* that solves (1.1) with a prescribed crack evolution $t\mapsto \Gamma _t$, we determine the pairs displacement-crack which satisfy the Griffith energy-dissipation balance. Finally, we select the “right” crack evolution according to some maximal dissipation principle.

In the easiest case of a pure elastic material, the system  (1.1) is coupled with the following constitutive law involving the Cauchy stress and the strain tensors:

1.2 $$\begin{aligned} \sigma (t)={\mathbb {C}}eu(t)\quad \text {in}\ \Omega \setminus \Gamma _t, t\in [0,T], \end{aligned}$$

where ${\mathbb {C}}$ is the elasticity tensor, which is fourth-order positive definite on the space of symmetric matrices ${\mathbb {R}}^{d\times d}_{\textrm{sym}}$, and $eu=\frac{1}{2}(\nabla u+\nabla u^T)$ is the strain tensor. In this setting, the Griffith criterion reads as

1.3 $$\begin{aligned}{} & {} \frac{1}{2}\Vert \dot{u}(t)\Vert _2^2+\frac{1}{2}\Vert eu(t)\Vert _2^2+{\mathcal {H}}^{d-1}(\Gamma _t\setminus \Gamma _0)\nonumber \\ {}{} & {} \quad =\frac{1}{2}\Vert \dot{u}(0)\Vert _2^2+\frac{1}{2}\Vert eu(0)\Vert _2^2 +\text{ work } \text{ of } \text{ external } \text{ forces } \end{aligned}$$

for all $t\in [0,T]$. We point out that the first two terms in the left-hand side of the above identity correspond to the mechanical energy (the sum of kinetic and elastic energy), while the term ${\mathcal {H}}^{d-1}(\Gamma _t\setminus \Gamma _0)$ models the energy used to increase the crack from $\Gamma _0$ to $\Gamma _t$.

In the literature, we can find several mathematical results for the model associated with (1.1) and (1.2). As for the existence of a solution when the evolution $t\mapsto \Gamma _t$ is prescribed, we refer to [10, 13] for the antiplane case, that is when $u(t):\Omega \setminus \Gamma _t\rightarrow {\mathbb {R}}$ is a scalar function and *eu* is replaced by $\nabla u$, and [4, 26] for the general case. Regarding the determination of the crack evolution $t\mapsto \Gamma _t$, we have only partial results. For example, we cite [5], where the authors characterize in the antiplane case and for $d=2$ the pairs displacement-crack which satisfy the energy-dissipation balance, and [11, 12] in which for $d=2$ the authors study the coupled problem under a suitable notion of maximal dissipation.

Viscoelastic materials, which exhibit both viscous and elastic behaviors when undergoing deformations, are another class widely studied in the literature. One of the simplest mathematical model is the Kelvin–Voigt one, where the constitutive law between the Cauchy stress and the strain tensors reads as

1.4 $$\begin{aligned}{} & {} \frac{1}{2}\Vert \dot{u}(t)\Vert _2^2+\frac{1}{2}\Vert eu(t)\Vert _2^2+{\mathcal {H}}^{d-1}(\Gamma _t\setminus \Gamma _0)\nonumber \\ {}{} & {} \quad =\frac{1}{2}\Vert \dot{u}(0)\Vert _2^2+\frac{1}{2}\Vert eu(0)\Vert _2^2 +\text{ work } \text{ of } \text{ external } \text{ forces } \end{aligned}$$

where ${\mathbb {C}}$ and ${\mathbb {B}}$ are the elasticity and the viscosity tensors, respectively. For the Kelvin–Voigt model, the Griffith criterion leads to the following energy-dissipation balance

1.5 $$\begin{aligned}{} & {} \frac{1}{2}\Vert \dot{u}(t)\Vert _2^2+\frac{1}{2}\Vert eu(t)\Vert _2^2+{\mathcal {H}}^{d-1}(\Gamma _t\setminus \Gamma _0)+\int _0^t\Vert e\dot{u}(s)\Vert _2^2\,\mathrm ds \nonumber \\{} & {} \quad =\frac{1}{2}\Vert \dot{u}(0)\Vert _2^2+\frac{1}{2}\Vert eu(0)\Vert _2^2+\text {work of external forces} \end{aligned}$$

for all $t\in [0,T]$. Notice that, with respect to formula (1.3), in (1.5) we need to take into account also the energy dissipated by the viscous term, which is given by $\int _0^t\Vert e\dot{u}(s)\Vert _2^2\,\mathrm ds$.

In [10, 26], we can find existing results for the linear viscoelastic problem (1.1) and (1.4), when the evolution of the crack is prescribed. Unfortunately, in those papers, it is also shown that the Griffith energy-dissipation balance (1.5) holds without the term ${\mathcal {H}}^{d-1}(\Gamma _t\setminus \Gamma _0)$. As a consequence, there is no pair displacement-crack which satisfies (1.5), unless the crack does not grow in time, i.e., $\Gamma _t=\Gamma _0$ for all $t\in [0,T]$. This phenomenon, which says that in the linear Kelvin–Voigt model the crack can not propagate, is well-known in mechanics as the *viscoelastic paradox*, see for instance [25, Chapter 7]. We point out that, if the viscosity tensor ${\mathbb {B}}$ is allowed to degenerate in a neighborhood of the moving crack, the viscoelastic paradox does not occur, as shown in [6]. For other versions of linear constitutive laws in the framework of viscoelastic materials, we refer for example to [7–9, 23].

More recently, viscoelastic materials in which the constitutive relation is nonlinear and given in an implicit form have been also considered. For example, in [3], the authors study the following elastodynamic system in a domain without cracks:

1.6 $$\begin{aligned} \ddot{u}(t)-\mathop {\textrm{div}}\nolimits (\sigma (t))=f(t)\quad \text {in}\ \Omega , t\in [0,T], \end{aligned}$$

with the implicit constitutive law

1.7 $$\begin{aligned} G(\sigma (t))=eu(t)+e\dot{u}(t)\quad \text {in}\ \Omega , t\in [0,T], \end{aligned}$$

where $G:{\mathbb {R}}^{d\times d}_{sym}\rightarrow {\mathbb {R}}^{d\times d}_{sym}$ is a nonlinear monotone operator which satisfies suitable *p*-growth assumptions. In particular, the prototypical models studied are

1.8 $$\begin{aligned} G_1(\xi ):=|\xi |^{p-2}\xi \quad \text {for}\ p>1,\qquad G_2(\xi )=\frac{\xi }{(1+|\xi |^a)^\frac{1}{a}}\quad \text {for}\ p=1, \text {with}\ a>0.\nonumber \\ \end{aligned}$$

As explained by Bulíček, Patel, Süli, and Şengül in their paper [3] (see also [21]), linear models may be inaccurate to describe real phenomena, while implicit constitutive theories allow for a more general structure in modeling than explicit ones. Moreover, as shown by Rajagopal in [22], the nonlinear relationship between the stress and the strain can be obtained after linearizing the strain, and so it make sense to consider implicit constitutive relations in the contest of small deformations. Under suitable assumptions on the initial data and on the nonlinear term *G*, Bulíček, Patel, Süli, and Şengül in [3] prove existence and uniqueness of solutions to the problem (1.6) and (1.7) via the Galerkin approximation.

The aim of our paper is to study the model of viscoelastic materials with implicit constitutive law of [3], in the framework of dynamic crack propagation. More precisely, we consider the elastodynamics system (1.1) with the constitutive relation

1.9 $$\begin{aligned} G(\sigma (t))=eu(t)+e\dot{u}(t)\quad \text {in}\ \Omega \setminus \Gamma _t, t\in [0,T], \end{aligned}$$

where $G:{\mathbb {R}}^{d\times d}_{sym}\rightarrow {\mathbb {R}}^{d\times d}_{sym}$ is a nonlinear monotone operator which satisfies suitable *p*-growth assumptions (more precisely (G1)–(G3) in Sect. 2). Since the linear growth $p=1$ is hard to handle even in the case with no cracks, we restrict ourselves to the range $p\in (1,2^*)$, where $2^*:=\frac{2d}{d-2}$ is the Sobolev critical exponent. The condition $p<2^*$, which also appears in [3], is needed to ensure that the displacement *u*(*t*) is an element of $L^2(\Omega \setminus \Gamma _t;{\mathbb {R}}^d)$. Indeed, from (1.9), we easily deduce that *u*(*t*) lives in the Sobolev space $W^{1,p'}(\Omega \setminus \Gamma _t;{\mathbb {R}}^d)$, being $p'=\frac{p}{p-1}$ the Hölder conjugate exponent of *p*, which is compactly embedded in $L^2(\Omega \setminus \Gamma _t;{\mathbb {R}}^d)$ whenever $p<2^*$. This simplifies the mathematical formulation of the problem. An interesting question, which is out of the scope of this paper, is whether this condition can be removed.

Our first result is Theorem 2.8, where we prove the existence of a solution to the problem (1.1) and (1.9) when the crack evolution $t\mapsto \Gamma _t$ is prescribed, under suitable conditions on the data and on the nonlinear term *G*. The proof of Theorem 2.8 follows the main ideas of [3], adapted to our setting. First, since the Galerkin approximation does not fit well with the framework of time-dependent domains, we use the discretization-in-time scheme exploited in [10]. Moreover, since we want to consider nonlinear operators which are not strictly monotone, we regularize *G* in order to invert the relation (1.9). This allows us to write the Cauchy tensor in terms of the displacement and to switch from the formulation (1.1) and (1.9) to a simpler system. More precisely, we fix $n\in {\mathbb {N}}$ and we search a discrete-in-time approximate solution to (1.1) and (1.9) with *G* replaced by its regularization. Then, we perform a discrete energy estimate (see Lemma 3.3), which allows us to pass to the limit as $n\rightarrow \infty$ to obtain a pair $(u,\sigma )$ which solves (1.1). We prove that the displacement *u* is more regular in time, and by using a standard technique in the monotone operator theory, we show that $(u,\sigma )$ satisfies also the implicit constitutive relation (1.9). We conclude this part of the paper with Theorem 3.10, where we prove that there is at most one pair $(u,\sigma )$ with the same regularity of the solution of Theorem 2.8 that solves (1.1) and (1.9) for a prescribed crack evolution $t\mapsto \Gamma _t$.

In the second part of the paper, we aim to study the validity of the Griffith energy-dissipation balance for the implicit nonlinear model (1.1) and (1.9). At first, in Theorem 4.1 we prove that the mechanical energy of every regular solution to problem (1.1) and (1.9) (in particular, of the one found in Theorem 2.8) satisfies the implicit energy balance

$$\begin{aligned} \frac{1}{2}\Vert \dot{u}(t)\Vert _2^2+\int _0^t\int _\Omega \sigma (s,x)\cdot e\dot{u}(s,x)\,\mathrm dx\,\mathrm ds= \frac{1}{2}\Vert \dot{u}(0)\Vert _2^2+ \text {work of external forces} \end{aligned}$$

for every $t\in [0,T]$. Then, we consider the strictly monotone operator $G(\xi )=|\xi |^{p-2}\xi$, so that our problem reduces to the nonlinear Kelvin–Voigt system

1.10 $$\begin{aligned} \ddot{u}(t)-\mathop {\textrm{div}}\nolimits (|eu(t)+e\dot{u}(t)|^{p'-2}(eu(t)+e\dot{u}(t)))=f(t)\quad \text {in}\ \Omega \setminus \Gamma _t, t\in [0,T].\nonumber \\ \end{aligned}$$

In this setting, the Griffith energy-dissipation balance takes the form

1.11 $$\begin{aligned}{} & {} \frac{1}{2}\Vert \dot{u}(t)\Vert _2^2+\frac{1}{p'}\Vert eu(t)\Vert _{p'}^{p'}+{\mathcal {H}}^{d-1}(\Gamma _t\setminus \Gamma _0)\nonumber \\ {}{} & {} \qquad +\int _0^t\int _\Omega |eu(s,x)+e\dot{u}(s,x)|^{p'-2}(eu(s,x)+e\dot{u}(s,x))\cdot e\dot{u}(s,x)\,\mathrm dx\,\mathrm ds\nonumber \\ {}{} & {} \qquad -\int _0^t\int _\Omega |eu(s,x)|^{p'-2}eu(s,x) \cdot e\dot{u}(s,x)\,\mathrm dx\,\mathrm ds\nonumber \\ {}{} & {} \quad =\frac{1}{2}\Vert \dot{u}(0)\Vert _2^2+\frac{1}{p'}\Vert eu(0)\Vert _{p'}^{p'}+\text{ work } \text{ of } \text{ external } \text{ forces } \end{aligned}$$

for every $t\in [0,T]$. In particular, the energy dissipated by the viscous term is given by

$$\begin{aligned}{} & {} \int _0^t\int _\Omega |eu(s,x)+e\dot{u}(s,x)|^{p'-2}(eu(s,x)+e\dot{u}(s,x))\cdot e\dot{u}(s,x)\,\mathrm dx\,\mathrm ds \\ {}{} & {} \quad -\int _0^t\int _\Omega |eu(s,x)|^{p'-2}eu(s,x)\cdot e\dot{u}(s,x)\,\mathrm dx\,\mathrm ds\ge 0, \end{aligned}$$

which reduces to the corresponding term in (1.5) for $p=2$ (notice that this term is nonnegative due to the monotonicity of $G^{-1}(\eta ):=|\eta |^{p'-2}\eta$). For this particular choice of *G*, in Corollary 4.3 we derive that the energy dissipation balance proved in Theorem 4.1 can be rewritten just in terms of the displacement *u* as (4.7). Therefore, the pair displacement-crack given by Theorem 2.8 satisfies (1.11) if and only if $\Gamma _t=\Gamma _0$ for every $t\in [0,T]$, i.e., when the crack does not grow in time. This shows that also the nonlinear Kelvin–Voigt model of dynamic fracture exhibits the viscoelastic paradox, as it happens in [10, 26] for the corresponding linear model.

We conclude the introduction by observing that the corresponding phase-field model of dynamic crack propagation has been analyzed in [20] (see also [21]). This is the one in which, roughly speaking, for a fixed $\epsilon >0$ the crack set is replaced by a function $v_\epsilon$ which is 0 in a $\epsilon$-neighborhood of the crack and 1 far from it. More precisely, in [20] the author proved that there exists a pair $(u_\epsilon ,v_\epsilon )$ which satisfies the elastodynamics system with the implicit constitutive law and the Griffith energy-dissipation balance for both the nonlinearities in (1.8). Therefore, it could be interesting to understand in a future paper if there is a connection between these two models and, in particular, if the viscoelastic paradox can also occur in the phase-field setting.

The rest of the paper goes as follows: in Sect. 2 we introduce the mathematical framework of our model of dynamic fracture for viscoelastic material, and we fix the main assumptions on the reference set, the crack evolution, and the nonlinearity *G* in the constitutive law. Moreover, in Definition 2.3 we give the notion of (weak) solution to problem (1.1) and (1.9), and we state our main existence result, which is Theorem 2.8. In Sect. 3 we prove Theorem 2.8 by performing a discretization-in-time scheme together with a regularization of the nonlinearity *G*. At first, we find an approximate solution in each node of the discretization of the regularized model. Then, in Lemma 3.3 we prove a discrete energy estimate, which allows us to pass to the limit when the parameter of the discretization and regularization goes to 0. Finally, we show that under suitable regularity assumptions the solution is unique. We conclude the paper with Sect. 4, where we prove that every regular solution to (1.1) and (1.9) satisfies the energy-dissipation identity of Theorem 4.1. Afterwards, we consider the nonlinear Kelvin–Voigt system (1.10), and we use the energy-dissipation identity to show that this model exhibits the viscoelastic paradox.

## Notation and formulation of the model

### Notation

The space of $m\times d$ matrices with real entries is denoted by ${\mathbb {R}}^{m\times d}$; in case $m=d$, the subspace of symmetric matrices is denoted by ${\mathbb {R}}^{d\times d}_{sym}$. For any $A,B\in {\mathbb {R}}^{d\times d }$ we denote with $A\cdot B$ the Frobenius scalar product, namely $A\cdot B:=\mathop {\textrm{Tr}}\nolimits (A^TB)$. Given a function $u:{\mathbb {R}}^d\rightarrow {\mathbb {R}}^m$, we denote its Jacobian matrix by $\nabla u$, whose components are $(\nabla u)_{ij}:= \partial _j u_i$ for $i\in \{1,\dots ,m\}$ and $j\in \{1,\dots ,d\}$; when $u:{\mathbb {R}}^d\rightarrow {\mathbb {R}}^d$, we use *eu* to denote the symmetric part of the gradient, namely $eu:=\frac{1}{2}(\nabla u+\nabla u^T)$. Given a tensor field $A:{\mathbb {R}}^d\rightarrow {\mathbb {R}}^{m\times d}$, by $\mathop {\textrm{div}}\nolimits A$ we mean its divergence with respect to rows, namely $(\mathop {\textrm{div}}\nolimits A)_i:= \sum _{j=1}^d\partial _jA_{ij}$ for $i\in \{1,\dots ,m\}$.

We denote the *d*-dimensional Lebesgue measure by ${\mathcal {L}}^d$ and the $(d-1)$-dimensional Hausdorff measure by ${\mathcal {H}}^{d-1}$; given a bounded open set $\Omega$ with Lipschitz boundary, by $\nu$ we mean the outer unit normal vector to $\partial \Omega$, which is defined ${\mathcal {H}}^{d-1}$-a.e. on the boundary. The Lebesgue and Sobolev spaces on $\Omega$ are defined as usual; the boundary values of a Sobolev function are always intended in the sense of traces. When there is no ambiguity, we simply write $\Vert \cdot \Vert _p$ to denote the norm in $L^p(\Omega ;{\mathbb {R}}^k)$ for all $p\in [1,\infty ]$ and $k\in {\mathbb {N}}$.

The norm of a generic Banach space *X* is denoted by $\Vert \cdot \Vert _X$; when *X* is a Hilbert space, we use $(\cdot ,\cdot )_X$ to denote its scalar product. We denote by $X'$ the dual of *X* and by $\langle \cdot ,\cdot \rangle _{X'}$ the duality product between $X'$ and *X*. Given two Banach spaces $X_1$ and $X_2$, the space of linear and continuous maps from $X_1$ to $X_2$ is denoted by ${\mathscr {L}}(X_1;X_2)$; given ${\mathbb {A}}\in {\mathscr {L}}(X_1;X_2)$ and $u\in X_1$, we write ${\mathbb {A}} u\in X_2$ to denote the image of *u* under ${\mathbb {A}}$.

Given an open interval $(a,b)\subset {\mathbb {R}}$ and $q\in [1,\infty ]$, we denote by $L^q(a,b;X)$ the space of $L^q$ functions from (*a*, *b*) to *X*; we use $W^{k,q}(a,b;X)$ to denote the Sobolev space of functions from (*a*, *b*) to *X* with derivatives up to order *k* in $L^q(a,b;X)$. Given $u\in W^{1,q}(a,b;X)$, we denote by $\dot{u}\in L^q(a,b;X)$ its derivative in the sense of distributions. When dealing with an element $u\in W^{1,q}(a,b;X)$ we always assume *u* to be the continuous representative of its class, and therefore, the pointwise value *u*(*t*) of *u* is well defined for all $t\in [a,b]$. We use $C_w^0([a,b];X)$ to denote the set of weakly continuous functions from [*a*, *b*] to *X*, namely the collection of maps $u:[a,b]\rightarrow X$ such that $t\mapsto \langle x',u(t)\rangle _{X'}$ is continuous from [*a*, *b*] to ${\mathbb {R}}$, for all $x'\in X'$.

### Mathematical framework

Let $T>0$ and $d\in {\mathbb {N}}$ with $d\ge 2$. Let $\Omega \subset {\mathbb {R}}^d$ be a bounded open set (which represents the reference configuration of the body) with Lipschitz boundary. Let $\partial _D\Omega$ be a Borel subset of $\partial \Omega$, on which we prescribe the Dirichlet condition, $\partial _N\Omega$ its complement in $\partial \Omega$, and $\Gamma \subset {{\overline{\Omega }}}$ the prescribed crack path. As in [6, 7], we assume the following hypotheses on the geometry of the crack and the Dirichlet part of the boundary:

1. $\Gamma$ is a closed set with ${\mathcal {L}}^d(\Gamma )=0$ and ${\mathcal {H}}^{d-1}(\Gamma \cap \partial \Omega )=0$;
2. $\Omega \setminus \Gamma$ is the union of two disjoint bounded open sets $\Omega _1$ and $\Omega _2$ with Lipschitz boundary;
3. $\partial _D\Omega \cap \partial \Omega _i$ contains the graph of a Lipschitz function $\theta _i$ over a non-empty open subset of ${\mathbb {R}}^{d-1}$ for all $i\in \{1,2\}$;
4. $\{\Gamma _t\}_{t\in [0,T]}$ is a family of closed subsets of $\Gamma$ satisfying $\Gamma _s\subseteq \Gamma _t$ for all $0\le s\le t\le T$.

We recall that the set $\Gamma _t$ represents the prescribed crack at time $t\in [0,T]$ inside $\Omega$.

Thanks to (E1)–(E4) for all $q\in [1,\infty ]$ the space $L^q(\Omega \setminus \Gamma _t;{\mathbb {R}}^d)$ coincides with $L^q(\Omega ;{\mathbb {R}}^d)$ for all $t\in [0,T]$. In particular, we can extend a function $u\in L^q(\Omega \setminus \Gamma _t;{\mathbb {R}}^d)$ to a function in $L^q(\Omega ;{\mathbb {R}}^d)$ by setting $u=0$ on $\Gamma _t$. Moreover, for all $q\in [1,\infty )$ the trace of $u\in W^{1,q}(\Omega {\setminus }\Gamma ;{\mathbb {R}}^d)$ is well defined on $\partial \Omega$ and there exists a constant $C_{tr}>0$, depending on $\Omega$, $\Gamma$, and *q*, such that

2.1 $$\begin{aligned} \Vert u\Vert _{L^q(\partial \Omega ;{\mathbb {R}}^d)}\le C_{tr}\Vert u\Vert _{W^{1,q}(\Omega \setminus \Gamma ;{\mathbb {R}}^d)}\quad \text {for all }u\in W^{1,q}(\Omega \setminus \Gamma ;{\mathbb {R}}^d). \end{aligned}$$

Hence, we can define the space

$$\begin{aligned} W^{1,q}_D(\Omega \setminus \Gamma ;{\mathbb {R}}^d):=\{u\in W^{1,q}(\Omega \setminus \Gamma ;{\mathbb {R}}^d):\, u=0\text { on }\partial _D\Omega \}. \end{aligned}$$

Furthermore, by using the second Korn inequality in $\Omega _1$ and $\Omega _2$ (see, e.g., [19, Theorem 2.4]) and taking the sum we can find a positive constant $C_K$, depending on $\Omega$, $\Gamma$, and *q*, such that

2.2 $$\begin{aligned} \Vert \nabla u\Vert _{L^q(\Omega ;{\mathbb {R}}^{d\times d})}\le {}&{} C_K(\Vert u\Vert _{L^q(\Omega ;{\mathbb {R}}^d)}^q+\Vert eu\Vert _{L^q(\Omega ;{\mathbb {R}}^{d\times d}_{sym})}^q)^{\frac{1}{q}}\nonumber \\ {}&{}\quad \text{ for } \text{ all } u\in W^{1,q}(\Omega \setminus \Gamma ;{\mathbb {R}}^d). \end{aligned}$$

Similarly, thanks to the Korn-Poincaré inequality (see, e.g., [19, Theorem 2.7]) we obtain also the existence of a constant $C_{KP}$, depending on $\Omega$, $\Gamma$, *q*, and $\partial _D\Omega$, such that

2.3 $$\begin{aligned} \Vert u\Vert _{W^{1,q}(\Omega \setminus \Gamma ;{\mathbb {R}}^d)}\le C_{KP}\Vert eu\Vert _{L^q(\Omega ;{\mathbb {R}}^{d\times d}_{sym})}\quad \text {for all}\ u\in W^{1,q}_D(\Omega \setminus \Gamma ;{\mathbb {R}}^d). \end{aligned}$$

Finally, for all $q\in (\frac{2d}{d+2},\infty ]$ the embedding $W^{1,q}(\Omega {\setminus }\Gamma ;{\mathbb {R}}^d)\hookrightarrow L^2(\Omega ;{\mathbb {R}}^d)$ is continuous and compact.

We fix $p\in (1,2^*)$, where $2^*$ is the Sobolev conjugate of 2, defined as

$$\begin{aligned} 2^*:={\left\{ \begin{array}{ll} \infty &{}\text {for}\ d=2,\\ \frac{2d}{d-2}&{}\text {for}\ d>2. \end{array}\right. } \end{aligned}$$

Notice that $p\in (1,2^*)$ if and only if $p'\in (\frac{2d}{d+2},\infty )$, where $p':=\frac{p}{p-1}$ is the Hölder conjugate exponent of *p*. We set $H:=L^2(\Omega ;{\mathbb {R}}^d)$ and we define the following spaces

$$\begin{aligned} V:=W^{1,p'}(\Omega \setminus \Gamma ;{\mathbb {R}}^d)\quad \text { and }\quad V_t:= W^{1,p'}(\Omega \setminus \Gamma _t;{\mathbb {R}}^d)\quad \text {for all}\ t\in [0,T]. \end{aligned}$$

We point out that in the definition of *V* and $V_t$, we are considering only the distributional gradient of *u* in $\Omega \setminus \Gamma$ and in $\Omega \setminus \Gamma _t$, respectively, and not the one in $\Omega$. Taking into account (2.2), we shall use on the set $V_t$ (and also on the set *V*) the equivalent norm

$$\begin{aligned} \Vert u\Vert _{V_t}:=\left( \Vert u\Vert _{p'}^{p'}+\Vert eu\Vert _{p'}^{p'}\right) ^{\frac{1}{{p'}}}\quad \text {for all }u\in V_t. \end{aligned}$$

Furthermore, by (2.1), we can consider the sets

$$\begin{aligned} V^D:=\{u\in V:\,u=0\text { on }\partial _D\Omega \},\qquad V_t^D:=\{u\in V_t:\,u=0\text { on }\partial _D\Omega \}\quad \text {for all}\ t\in [0,T], \end{aligned}$$

which are closed subspaces of *V* and $V_t$, respectively.

#### Remark 2.1

Since $p\in (1,2^*)$, by exploiting (E1)–(E4) we derive that for all $t\in [0,T]$ the space $V_t^D$ is a separable reflexive Banach space with embedding

$$\begin{aligned} V_t^D\hookrightarrow H\text { continuous, compact, and dense}. \end{aligned}$$

In particular, the aforementioned condition on *p* is used to deduce the compactness of $V_t^D$ in *H*. Therefore, the embedding $H\hookrightarrow (V_t^D)'$, which is defined by

2.4 $$\begin{aligned} \langle h,u\rangle _{(V_t^D)'}:=(h,u)_H\quad \text {for}\ h\in H\ \text {and}\ u\in V_t^D, \end{aligned}$$

is continuous, and the same holds true also for $V_t$, *V*, and $V^D$.

Let us consider a nonlinear operator $G:{\mathbb {R}}^{d\times d}_{sym}\rightarrow {\mathbb {R}}^{d\times d}_{sym}$ satisfying the following assumptions:

1. there exists a convex function $\phi :{\mathbb {R}}^{d\times d}_{sym}\rightarrow {\mathbb {R}}$ of class $C^1$ such that $G(\xi )=\nabla \phi (\xi )$ for all $\xi \in {\mathbb {R}}^{d\times d}_{sym}$;
2. there exist constants $b_1>0$ and $b_2\ge 0$ such that $G(\xi )\cdot \xi \ge b_1|\xi |^p-b_2$ for all $\xi \in {\mathbb {R}}^{d\times d}_{sym}$;
3. there exists a constant $b_3> 0$ such that $|G(\xi )|\le b_3(1+|\xi |^{p-1})$ for all $\xi \in {\mathbb {R}}^{d\times d}_{sym}$.

#### Remark 2.2

The assumption (G1) implies that *G* is continuous and monotone, i.e.,

2.5 $$\begin{aligned} (G(\xi _1)-G(\xi _2))\cdot (\xi _1-\xi _2)\ge 0\quad \text {for all}\ \xi _1,\xi _2\in {\mathbb {R}}^{d\times d}_{sym}. \end{aligned}$$

Moreover, up to add a constant, we always assume that $\phi (0)=0$.

Given

1. $$f\in L^2(0,T;H)$$;
2. $$z\in W^{2,p'}(0,T;V_0)\cap W^{2,2}(0,T;H)$$;
3. $u^0,u^1\in V_0$ such that $u^0-z(0)\in V_0^D$ and $u^1-{\dot{z}}(0)\in V_0^D$;

we study the following dynamic viscoelastic system with implicit nonlinear constitutive law:

2.6 $$\begin{aligned} {\left\{ \begin{array}{ll} \ddot{u}(t)-\mathop {\textrm{div}}\nolimits (\sigma (t))=f(t)&{}\text {in}\ \Omega \setminus \Gamma _t, t\in [0,T],\\ G(\sigma (t))=eu(t)+e\dot{u}(t)&{}\text {in}\ \Omega \setminus \Gamma _t, t\in [0,T], \end{array}\right. } \end{aligned}$$

equipped with the boundary conditions

2.7 $$\begin{aligned}{} & {} u(t)=z(t) \quad \text {on}\ \partial _D\Omega , \quad t\in [0,T], \end{aligned}$$

2.8 $$\begin{aligned}{} & {} \sigma (t)\nu =0 \quad \text {on}\ \partial _N\Omega \cup \Gamma _t, \quad t\in [0,T], \end{aligned}$$

where $\nu$ denotes the outward unit normal to $\partial \Omega$, and the initial conditions

2.9 $$\begin{aligned}&u(0)=u^0,\quad {\dot{u}}(0)=u^1 \quad \text {in}\ \Omega \setminus \Gamma _0. \end{aligned}$$

Notice that in (2.6)–(2.9) the explicit dependence on *x* is omitted to enlighten notation. As usual, the Neumann boundary conditions are only formal, and their meaning will be explained in Remark 2.4.

From now on we always assume that $p\in (1,2^*)$ and that (E1)–(E4), (G1)–(G3), and (D1)–(D3) are satisfied. Let us define the following functional spaces:

$$\begin{aligned}&{\mathcal {V}}:=\{\varphi \in W^{1,p'}(0,T;V)\cap W^{1,\infty }(0,T;H)\,:\,\varphi (t)\in V_t\text { for all}\ t\in [0,T]\},\\&{\mathcal {D}}:=\{\varphi \in C^1_c(0,T;V)\,:\,\varphi (t)\in V_t^D\text { for all}\ t\in [0,T]\}. \end{aligned}$$

Similarly to [14], we introduce the following notion of weak solution.

#### Definition 2.3

(Weak solution) A pair $(u,\sigma )\in {\mathcal {V}}\times L^p(0,T;L^p(\Omega ,{\mathbb {R}}^{d\times d}_{sym}))$ is a *weak solution* to the nonlinear viscoelastic system (2.6)–(2.8) if

(i) $$u(t)-z(t)\in V_t^D$$ for all $$t\in [0,T]$$;
(ii) the following identity holds
  2.10 $$\begin{aligned}{} & {} -\int _0^T(\dot{u}(t),{{\dot{\varphi }}}(t))_H\,\mathrm dt+\int _0^T(\sigma (t),e \varphi (t))_{p,p'}\,\mathrm dt\nonumber \\ {}{} & {} \quad =\int _0^T( f(t),\varphi (t))_H\,\mathrm dt\quad \text{ for } \text{ all }\ \varphi \in {\mathcal {D}} \end{aligned}$$
  where $(g,h)_{p,p'}:=\int _\Omega g(x)\cdot h(x)\,\mathrm dx$ for all $g\in L^p(\Omega ;{\mathbb {R}}^{d\times d}_{sym})$ and $h\in L^{p'}(\Omega ;{\mathbb {R}}^{d\times d}_{sym})$;
(iii) the constitutive law
  2.11 $$\begin{aligned} G(\sigma (t))=eu(t)+e\dot{u}(t)\quad \text {in}\ \Omega \setminus \Gamma _t\ \text {for a.e.}\ t\in [0,T] \end{aligned}$$
  is satisfied.

#### Remark 2.4

The Neumann boundary conditions (2.8) are formally used to pass from the strong formulation (2.6)–(2.8) to the weak formulation (2.10). Notice that, if *u*(*t*), $\sigma (t)$, and $\Gamma _t$ are sufficiently regular, then (2.8) can be deduced from (2.10) by using integration by parts in space.

We want to give a meaning to the initial conditions (2.9) for a weak solution $(u,\sigma )$ to (2.6)–(2.8). To this aim, we first recall the following result (see, for instance [15, Chapitre XVIII, §5, Lemme 6]).

#### Lemma 2.5

Let *X*, *Y* be reflexive Banach spaces such that $X\hookrightarrow Y$ continuously. Then,

$$\begin{aligned} L^{\infty }(0, T;X)\cap C^0_w([0, T];Y)= C^0_w([0, T];X). \end{aligned}$$

Moreover, we need the following regularity result for the weak solutions to (2.6)–(2.8).

#### Lemma 2.6

Let $(u,\sigma )\in {\mathcal {V}}\times L^p(0,T;L^p(\Omega ,{\mathbb {R}}^{d\times d}_{sym}))$ be a weak solution to the nonlinear viscoelastic system (2.6)–(2.8). Then, $u\in W^{2,q}(0,T;(V_0^D)')$, where $q:=\min \{2,p\}$. In particular $u\in C^0([0,T];V)$ and $\dot{u}\in C^0_w([0,T];H)$.

#### Proof

Let *q* be given as in the statement. We define $\Lambda \in L^q(0,T;(V^D_0)')$ as follows:

$$\begin{aligned} \langle \Lambda (t),v\rangle _{(V_0^D)'}:=-(\sigma (t),ev)_{p,p'}+(f (t),v)_H\quad \text {for all}\ v\in V^D_0\ \text {and for a.e.}\ t\in [0,T]. \end{aligned}$$

Let us consider a test function $\psi \in C^{1}_c(0,T)$, then for all $v\in V^D_0$ the function $\varphi (t):=\psi (t)v$ satisfies

2.12 $$\begin{aligned} \varphi \in C^{1}_c(0,T;V),\quad \varphi (t)\in V_0^D\subset V_t^D \quad \text {for all}\ t\in [0,T]. \end{aligned}$$

Thanks to (2.10), since $\varphi \in {\mathcal {D}}$ from (2.12), we can write

$$\begin{aligned} -\int _0^T(\dot{u}(t),v)_H{{\dot{\psi }}}(t)\,\mathrm dt&=-\int _0^T(\sigma (t),ev)_{p,p'}\psi (t)\,\mathrm dt+\int _0^T(f (t),v)_H\psi (t)\,\mathrm dt\\&=\int _0^T\langle \Lambda (t),v\rangle _{(V_0^D)'}\psi (t)\,\mathrm dt, \end{aligned}$$

which implies by (2.4)

$$\begin{aligned} \Big \langle -\int _0^T\dot{u}(t){{\dot{\psi }}}(t)\,\mathrm dt,v\Big \rangle _{(V_0^D)'}=\Big \langle \int _0^T \Lambda (t)\psi (t)\,\mathrm dt,v\Big \rangle _{(V_0^D)'}\quad \text {for all}\ v\in V^D_0. \end{aligned}$$

Hence, we get

2.13 $$\begin{aligned} -\int _0^T\dot{u}(t){{\dot{\psi }}}(t)\,\mathrm dt= \int _0^T \Lambda (t)\psi (t)\,\mathrm dt\quad \text {in}\ (V^D_0)'\ \text {for all}\ \psi \in C^{1}_c(0,T). \end{aligned}$$

Since $\dot{u}\in L^\infty (0,T;H)\hookrightarrow L^\infty (0,T;(V^D_0)')$ then identity (2.13) implies

$$\begin{aligned} u\in W^{2,q}(0,T;(V^D_0)'). \end{aligned}$$

Therefore $\dot{u}\in W^{1,q}(0,T;(V^D_0)')\hookrightarrow C^0([0,T];(V^D_0)')$. Since $\dot{u}\in L^{\infty }(0,T;H)$, by Lemma 2.5 we deduce that $\dot{u}\in C^0_w([0,T];H)$. Finally, we have $W^{1,p'}(0,T;V)\hookrightarrow C^0([0,T];V)$ hence $u\in C^0([0,T];V)$. $\square$

If $(u,\sigma )\in {\mathcal {V}}\times L^p(0,T;L^p(\Omega ;{\mathbb {R}}^{d\times d}_{sym}))$ is a weak solution to (2.6)–(2.8), then *u*(*t*) and $\dot{u}(t)$ are well defined as functions of *V* and *H*, respectively, for all $t\in [0,T]$. Therefore, it makes sense to evaluate them at time $t=0$ in order to make consistent the following definition.

#### Definition 2.7

(Initial conditions) We say that a weak solution $(u,\sigma )\in {\mathcal {V}}\times L^p(0,T;L^p(\Omega ;{\mathbb {R}}^{d\times d}_{sym}))$ to the nonlinear viscoelastic system (2.6)–(2.8) satisfies the initial conditions (2.9) if

$$\begin{aligned} u(0)=u^0\quad \text{ in }\ V,\quad \dot{u}(0)=u^1\quad \text{ in }\ H \end{aligned}$$

The main existence result of this paper is the following theorem.

#### Theorem 2.8

There exists a weak solution $(u,\sigma )\in {\mathcal {V}}\times L^p(0,T;L^p(\Omega ;{\mathbb {R}}^{d\times d}_{sym}))$ to the nonlinear viscoelastic system (2.6)–(2.8) satisfying the initial conditions (2.9). Moreover, $u\in W^{2,2}(0,T;H)$.

The proof of Theorem 2.8 is postponed to the next section. We point out that the displacement *u* of the solution found in Theorem 2.8 is more regular in time, more precisely $\ddot{u}\in L^2(0,T;H)$. This regularity is used at the end of Sect. 3 to prove a uniqueness result for the nonlinear viscoelastic system  (2.6)–(2.9). Moreover, we exploit such a regularity in Sect. 4 to show the energy-dissipation balance of Theorem 4.1. This identity implies the viscoelastic paradox, which is discussed at the end of the paper.

## Existence of solutions

This section is devoted to the proof of Theorem 2.8. As explained in the introduction, the main idea is to combine the discretization-in-time scheme of [10] with the regularization of the nonlinear operator *G* introduced in [3]. Therefore, we rephrase the system (2.6) in a simpler way, and we use Browder-Minty Theorem to find a sequence of approximate solutions in each node of the discretization scheme. Then in Lemma 3.3 we prove a discrete energy estimate and we use a compactness argument to obtain a pair $(u,\sigma )$ which solves (2.10) (see Lemma 3.8). Finally, in Lemma 3.9, by performing a standard argument in the theory of nonlinear monotone operators we show the validity of the constitutive law (2.11).

Let us fix $n\in {\mathbb {N}}$ and setWe define $G_n:{\mathbb {R}}^{d\times d}_{sym}\rightarrow {\mathbb {R}}^{d\times d}_{sym}$ as

$$\begin{aligned} G_n(\xi ):=G(\xi )+\frac{1}{n}|\xi |^{p-2}\xi \quad \text {for all}\ \xi \in {\mathbb {R}}^{d\times d}_{sym}. \end{aligned}$$

Notice that $G_n$ still satisfies (G1)–(G3) with $\phi$ replaced by

$$\begin{aligned} \phi _n(\xi ):=\phi (\xi )+\frac{1}{np}|\xi |^p\quad \text {for all}\ \xi \in {\mathbb {R}}^{d\times d}_{sym}, \end{aligned}$$

and with $b_3$ replaced by $b_3+1$. Since $G_n$ is strictly monotone, by the standard theory of monotone operators there exists the inverse operator $G_n^{-1}:{\mathbb {R}}^{d\times d}_{sym}\rightarrow {\mathbb {R}}^{d\times d}_{sym}$, which is still strictly monotone. Moreover, if we introduce the Legendre transform $\phi _n^*$ of $\phi _n$, defined as

$$\begin{aligned} \phi _n^*(\eta ):=\sup _{\xi \in {\mathbb {R}}^{d\times d}_{sym}}\{\eta \cdot \xi -\phi _n(\xi )\}\quad \text {for all}\ \eta \in {\mathbb {R}}^{d\times d}_{sym}, \end{aligned}$$

by (G1)–(G3) we have that $\phi _n^*:{\mathbb {R}}^{d\times d}_{sym}\rightarrow {\mathbb {R}}$ is still a convex function of class $C^1$ and $G_n^{-1}$ satisfies

3.1 $$\begin{aligned}&G_n^{-1}(\eta )=\nabla \phi _n^*(\eta ){} & {} \quad \text {for all}\ \eta \in {\mathbb {R}}^{d\times d}_{sym}, \end{aligned}$$

3.2 $$\begin{aligned}&G_n^{-1}(\eta )\cdot \eta \ge c_1|\eta |^{p'}- c_2{} & {} \quad \text {for all}\ \eta \in {\mathbb {R}}^{d\times d}_{sym}, \end{aligned}$$

3.3 $$\begin{aligned}&|G_n^{-1}(\eta )|\le c_3(1+|\eta |^{p'-1}){} & {} \quad \text {for all}\ \eta \in {\mathbb {R}}^{d\times d}_{sym}, \end{aligned}$$

for suitable constants $c_1,c_3>0$ and $c_2\ge 0$ independent of $n\in {\mathbb {N}}$. Furthermore, if we define $\eta _0:=G(0)=G_n(0)$, by the assumption $\phi (0)=0$ (see Remark 2.2) we have

$$\begin{aligned} \phi _n^*(\eta _0)=-\phi _n(0)=0. \end{aligned}$$

Therefore, thanks to the convexity of $\phi _n^*$ we derive

3.4 $$\begin{aligned}&\phi _n^*(\eta )\ge \phi _n^*(\eta _0)+G_n^{-1}(\eta _0)\cdot (\eta -\eta _0)=0\quad \text {for all}\ \eta \in {\mathbb {R}}^{d\times d}_{sym}, \end{aligned}$$

3.5 $$\begin{aligned}&\phi _n^*(\eta )\le \phi _n^*(\eta _0)+G_n^{-1}(\eta )\cdot (\eta -\eta _0)\le c_4(1+|\eta |^{p'})\quad \text {for all}\ \eta \in {\mathbb {R}}^{d\times d}_{sym}, \end{aligned}$$

for a suitable constant $c_4>0$ independent of $n\in {\mathbb {N}}$.

For all $k\in \{1,\dots ,n\}$ we search for a function $u_n^k\in V$ with $u_n^k-z_n^k\in V_n^k$ satisfying the following identity

3.6 $$\begin{aligned} (\delta ^2u_n^k,\varphi )_H+(G^{-1}_n(eu_n^k+e\delta u_n^k),e\varphi )_{p,p'}=(f_n^k,\varphi )_H\quad \text {for all}\ \varphi \in V_n^k, \end{aligned}$$

where

3.7 $$\begin{aligned} \delta u_n^k:=\frac{u_n^k-u_n^{k-1}}{\tau _n},\qquad \delta ^2 u_n^k:=\frac{\delta u_n^k-\delta u_n^{k-1}}{\tau _n}\quad \text {for}\ k\in \{1,\dots ,n\}. \end{aligned}$$

To this aim, we find a function $v_n^k\in V_n^k$ which solves

3.8 $$\begin{aligned}{} & {} (\delta ^2 v_n^k+\delta ^2 z_n^k,\varphi )_H+(G^{-1}_n(ev_n^k+e\delta v_n^k+ez_n^k+e\delta z_n^k),e\varphi )_{p,p'}\nonumber \\{} & {} \quad =(f_n^k,\varphi )_H\quad \text {for all}\ \varphi \in V_n^k, \end{aligned}$$

where $\delta z_n^k$ and $\delta ^2z_n^k$ are defined similarly to (3.7) starting from $z_n^k$. Indeed, the function $v_n^k\in V_n^k$ solves (3.8) if and only if $u_n^k:=v_n^k+z_n^k\in V$ satisfies $u_n^k-z_n^k=v_n^k\in V_n^k$ and (3.6).

To solve (3.8), we consider the family of nonlinear operators $F_n^k:V_n^k\rightarrow (V_n^k)'$ defined by

$$\begin{aligned} \langle F_n^k(v),\varphi \rangle _{(V_n^k)'}&:=\tfrac{1}{\tau _n^2}(v+v_n^{k-2}-2v_n^{k-1}+\tau _n^2\delta ^2z_n^k-\tau _n^2f_n^k,\varphi )_H\\&\quad +(G^{-1}_n((1+\tfrac{1}{\tau _n})ev-\tfrac{1}{\tau _n}v_n^{k-1}+ez_n^k+e\delta z_n^k),e\varphi )_{p,p'} \end{aligned}$$

for $v,\varphi \in V_n^k$. It is clear that $v_n^k\in V_n^k$ solves (3.8) if and only if

3.9 $$\begin{aligned} F_n^k(v_n^k)=0\quad \text {in}\ (V_n^k)'. \end{aligned}$$

To find a solution to (3.9) we need the following result, whose proof can be found in [2, 17].

### Theorem 3.1

(Browder-Minty) Let *X* be a reflexive Banach space and let $F:X\rightarrow X'$ be a monotone, hemicontinuous, and coercive operator. Then, *F* is surjective. Moreover, if *F* is strictly monotone, then *F* is also injective.

Let us show that $F_n^k$ satisfies the hypotheses of Theorem 3.1.

### Proposition 3.2

For every $n\in {\mathbb {N}}$ and $k\in \{1,\dots ,n\}$ the nonlinear operator $F_n^k:V_n^k\rightarrow (V_n^k)'$ is strictly monotone, coercive, and hemicontinuous.

### Proof

Let us fix $n\in {\mathbb {N}}$ and $k\in \{1,\dots ,n\}$. We start by proving that $F_n^k$ is a strictly monotone operator, i.e.,

$$\begin{aligned} \langle F_n^k(v)-F_n^k(w),v-w\rangle _{(V_n^k)'}>0 \quad \text {for all}\ v,w\in V_n^k\ \text {with}\ v\ne w. \end{aligned}$$

By the definition of $F_n^k$, for all $v,w\in V_n^k$ with $v\ne w$ we have

3.10 $$\begin{aligned} \langle F_n^k(v)-F_n^k(w),v-w\rangle _{(V_n^k)'}{} & {} =\frac{1}{\tau _n^2}\Vert v-w\Vert ^2_H+(G^{-1}_n(c_n ev+h_n^k) \nonumber \\ {}{} & {} \quad -G^{-1}_n(c_n ew+h_n^k),ev-ew)_{p,p'}, \end{aligned}$$

where

$$\begin{aligned} c_n:=1+\tfrac{1}{\tau _n}>0,\quad h_n^k:=-\tfrac{1}{\tau _n}ev_n^{k-1}+ez_n^k+e\delta z_n^k\in V_n^k. \end{aligned}$$

By using in (3.10) the monotonicity of $G_n^{-1}$ with $\eta _1=c_nev+h_n^k$ and $\eta _2=c_n ew+h_n^k$, we can write

$$\begin{aligned} \langle F_n^k(v)-F_n^k(w),v-w\rangle _{(V_n^k)'}\ge \frac{1}{\tau _n^2}\Vert v-w\Vert ^2_H>0, \end{aligned}$$

which shows the strictly monotonicity of $F_n^k$.

To prove the coerciveness of $F_n^k$, we have to show that

3.11 $$\begin{aligned} \frac{\langle F_n^k(v),v\rangle _{(V_n^k)'}}{\Vert v\Vert _{V_n^k}}\rightarrow \infty \quad \text {as}\ \Vert v\Vert _{V_n^k}\rightarrow \infty . \end{aligned}$$

Notice that

$$\begin{aligned} \langle F_n^k(v),v\rangle _{(V_n^k)'}&= d_n\Vert v\Vert _H^2 + d_n(\ell _n^k,v)_H \\&\quad +\frac{1}{c_n}(G^{-1}_n(c_n ev+h_n^k),c_n ev+h_n^k)_{p,p'}\\&\quad -\frac{1}{c_n}(G^{-1}_n(c_n ev+h_n^k),h_n^k)_{p,p'}, \end{aligned}$$

where

$$\begin{aligned} d_n:=\frac{1}{\tau _n^2}>0,\quad \ell _n^k:=v_n^{k-2}-2v_n^{k-1}+\tau _n^2\delta ^2z_n^k+\tau _n^2f_n^k\in H. \end{aligned}$$

Thanks to (3.2), (3.3), and Young inequality, for all $\varepsilon >0$ we have

3.12 $$\begin{aligned}&\frac{1}{c_n}(G^{-1}_n(c_n ev+h_n^k),c_n ev+h_n^k)_{p,p'}-\frac{1}{c_n}(G^{-1}_n(c_n ev+h_n^k),h_n^k)_{p,p'}\nonumber \\ {}&\quad \ge \frac{c_1}{c_n}\Vert c_n ev+h_n^k\Vert ^{p'}_{p'}-\frac{c_2}{c_n}{\mathcal {L}}^d(\Omega )-\frac{1}{c_n}\Vert G^{-1}_n(c_n ev+h_n^k)\Vert _{p}\Vert h_n^k\Vert _{p'}\nonumber \\ {}&\quad \ge \frac{c_1}{c_n}\Vert c_n ev+h_n^k\Vert ^{p'}_{p'}-\frac{c_2}{c_n}{\mathcal {L}}^d(\Omega )-\frac{\varepsilon ^p}{pc_n}\Vert G^{-1}_n(c_n ev+h_n^k)\Vert ^p_{p}-\frac{1}{p'c_n\varepsilon ^{p'}}\Vert h_n^k\Vert ^{p'}_{p'}\nonumber \\ {}&\quad \ge \frac{c_1}{c_n}\Vert c_n ev+h_n^k\Vert ^{p'}_{p'}-\frac{c_2}{c_n}{\mathcal {L}}^d(\Omega )-\frac{1}{p'c_n\varepsilon ^{p'}}\Vert h_n^k\Vert ^{p'}_{p'} \nonumber \\ {}&\qquad -\frac{\varepsilon ^p}{pc_n}(2^{p-1}c_3^p\Vert c_n ev+h_n^k\Vert ^{p'}_{p'}+2^{p-1}c_3^p {\mathcal {L}}^d(\Omega ))\nonumber \\ {}&\quad =\frac{1}{c_n}\Big (c_1-\frac{2^{p-1}c_3^p\varepsilon ^p}{p}\Big )\Vert c_n ev+h_n^k\Vert ^{p'}_{p'}\nonumber \\ {}&\qquad -\frac{1}{p'c_n\varepsilon ^{p'}}\Vert h_n^k\Vert ^{p'}_{p'}-\frac{1}{c_n}\Big (c_2+\frac{2^{p-1}c_3^p\varepsilon ^p}{p} \Big ){\mathcal {L}}^d(\Omega ). \end{aligned}$$

In particular, the Korn-Poincaré inequality (2.3) yields

$$\begin{aligned} \frac{c_n^{p'}}{C_{KP}^{p'}}\Vert v\Vert _{V_n^k}^{p'}\le \Vert c_n ev\Vert ^{p'}_{p'}\le 2^{p'-1}\Vert c_n ev+h_n^k\Vert ^{p'}_{p'}+2^{p'-1}\Vert h_n^k\Vert ^{p'}_{p'}. \end{aligned}$$

Hence, from (3.12) we deduce

3.13 $$\begin{aligned}&\frac{1}{c_n}(G^{-1}_n(c_n ev+h_n^k),c_n ev+h_n^k)_{p,p'}-\frac{1}{c_n}(G^{-1}_n(c_n ev+h_n^k),h_n^k)_{p,p'}\nonumber \\&\quad \ge \frac{c_n^{p'-1}}{2^{p'-1}C_{KP}^{p'}}\Big (c_1-\frac{2^{p-1}c_3^p\varepsilon ^p }{p}\Big )\Vert v\Vert ^{p'}_{V_n^k}-\frac{1}{c_n}\Big (c_1-\frac{2^{p-1}c_3^p\varepsilon ^p }{p}+\frac{1}{p'\varepsilon ^{p'}}\Big )\Vert h_n^k\Vert ^{p'}_{p'}\nonumber \\&\qquad -\frac{1}{c_n}\Big (c_2+\frac{2^{p-1}c_3^p\varepsilon ^p}{p} \Big ){\mathcal {L}}^d(\Omega ). \end{aligned}$$

By applying again Young inequality, we can write

3.14 $$\begin{aligned}&d_n\Vert v\Vert _H^2+d_n(\ell _n^k,v)_H\ge \frac{d_n}{2}\Vert v\Vert _H^2-\frac{d_n}{2}\Vert \ell _n^k\Vert _H^2. \end{aligned}$$

If we choose

$$\begin{aligned} 0<\varepsilon <\left( \frac{c_1 p}{2^{p-1}c_3^p}\right) ^{\frac{1}{p}}, \end{aligned}$$

thanks to (3.13) and (3.14) we obtain the existence a positive constant $K_1$ such that

3.15 $$\begin{aligned} \langle F_n^k(v),v\rangle _{(V_n^k)'} \ge K_1\left( \Vert v\Vert _H^2+\Vert v\Vert ^{p'}_{V_n^k}-\Vert h_n^k\Vert ^{p'}_{p'}-\Vert \ell _n^k\Vert _H^2-1\right) . \end{aligned}$$

Clearly, we have

3.16 $$\begin{aligned} \frac{\Vert h_n^k\Vert ^{p'}_{p'}+\Vert \ell _n^k\Vert _H^2+1}{\Vert v\Vert _{V_n^k}}\rightarrow 0\quad \text {as}\ \Vert v\Vert _{V_n^k}\rightarrow \infty . \end{aligned}$$

Moreover, we can write

3.17 $$\begin{aligned} \frac{\Vert v\Vert _H^2+\Vert v\Vert ^{p'}_{V_n^k}}{\Vert v\Vert _{V_n^k}}\ge \Vert v\Vert _{V_n^k}^{p'-1}\rightarrow \infty \quad \text {as}\ \Vert v\Vert _{V_n^k}\rightarrow \infty . \end{aligned}$$

Thanks to (3.15)–(3.17) we get (3.11).

To prove the hemicontinuity of $F_n^k$, we need to show that for all $u,v,w\in V_n^k$ there exists $t_0=t_0(u,v,w)$ such that the function $[-t_0,t_0]\ni t\mapsto \langle F_n^k(v+tu),w\rangle _{(V_n^k)'}$ is continuous in $t=0$. We fix $u,v,w\in V_n^k$ and we notice that

$$\begin{aligned} \langle F_n^k(v+tu),w\rangle _{(V_n^k)'}= & {} d_n(v+\ell _n^k,w)_H+d_nt(u,w)_H\\{} & {} +(G_n^{-1}(c_n(ev+teu)+h_n^k),ew)_{p,p'}. \end{aligned}$$

Moreover, we can write

3.18 $$\begin{aligned} G_n^{-1}(c_n(ev+teu)+h_n^k)\cdot ew\xrightarrow [t\rightarrow 0]{a.e.\ }G_n^{-1}(c_nev+h_n^k)\cdot ew, \end{aligned}$$

and thanks to (3.3) we get

3.19 $$\begin{aligned}&|(G_n^{-1}(c_n(ev+teu)+h_n^k),ew)_{p,p'}| \nonumber \\&\quad \le \frac{1}{p}\Vert G_n^{-1}(c_n(ev+teu)+h_n^k)\Vert _p^p+\frac{1}{p'}\Vert ew\Vert ^{p'}_{p'}\nonumber \\&\quad \le \frac{2^{p-1}c_3^p}{p}\Vert c_n(ev+teu)+h_n^k\Vert _{p'}^{p'}+\frac{2^{p-1}c_3^p}{p}{\mathcal {L}}^d(\Omega ) +\frac{1}{p'}\Vert ew\Vert ^{p'}_{p'}\nonumber \\&\quad \le K_2(\Vert c_n ev+h_n^k\Vert _{p'}^{p'}+\Vert eu\Vert _{p'}^{p'}+\Vert ew\Vert ^{p'}_{p'}+1), \end{aligned}$$

for a positive constant $K_2$. By using (3.18), (3.19), and dominate convergence theorem we obtain

3.20 $$\begin{aligned} (G_n^{-1}(c_n(ev+teu)+h_n^k), ew)_{p,p'}\xrightarrow [t\rightarrow 0]{}(G_n^{-1}(c_nev+h_n^k), ew)_{p,p'}. \end{aligned}$$

Since $d_nt(u,w)_H\rightarrow 0$ as $t\rightarrow 0$, by (3.20) we have

$$\begin{aligned}{} & {} \langle F_n^k(v+tu),w\rangle _{(V_n^k)'}\xrightarrow [t\rightarrow 0]{}d_n(v+\ell _n^k,w)_H+(G_n^{-1}(c_n ev+h_n^k),ew)_{p,p'}\\ {}{} & {} \quad =\langle F_n^k(v),w\rangle _{(V_n^k)'}, \end{aligned}$$

$$\square $$

Thanks to Theorem 3.1 and Proposition 3.2, we obtain that for all $n\in {\mathbb {N}}$ and $k\in \{1,\dots ,n\}$ the nonlinear operator $F_n^k:V_n^k\rightarrow (V_n^k)'$ is bijective, and hence there exists a unique $v_n^k\in V_n^k$ which solves (3.8). As a consequence, the function $u_n^k=v_n^k+z_n^k\in V$ is the unique solution to (3.6).

Let us define

3.21 $$\begin{aligned} \sigma _n^k:=G_n^{-1}(eu_n^k+e\delta u_n^k)\quad \text {for all}\ k\in \{1,\dots ,n\}. \end{aligned}$$

In the next lemma, we show a uniform energy estimate with respect to *n* for the family $\{(u_n^k,\sigma _n^k)\}_{k=1}^n$, which will be used to pass to the limit as $n\rightarrow \infty$ in the discrete equation (3.6).

### Lemma 3.3

There exists a positive constant $C_1$, independent of $n\in {\mathbb {N}}$, such that

3.22 $$\begin{aligned} \max _{i\in \{1,\dots ,n\}}\Vert u_n^i\Vert _V+\max _{i\in \{1,\dots ,n\}}\Vert \delta u_n^i\Vert _H+\sum _{i=1}^n \tau _n\Vert \delta u_n^i\Vert ^{p'}_{V}+\sum _{i=1}^n\tau _n\Vert \sigma _n^i\Vert ^{p}_{p}\le C_1. \end{aligned}$$

### Proof

We take $\varphi =\tau _n(u_n^k-z_n^k)\in V_n^k$ as a test function in (3.6). Therefore, we obtain

3.23 $$\begin{aligned}&\tau _n(G^{-1}_n(eu_n^k+e\delta u_n^k),e u_n^k-ez_n^k)_{p,p'}=\tau _n(f_n^k, u_n^k- z_n^k)_H-\tau _n(\delta ^2 u_n^k, u_n^k-z_n^k)_H. \end{aligned}$$

We fix $i\in \{1,\dots ,n\}$ and by summing in (3.23) over $k\in \{1,\dots ,i\}$ we obtain

3.24 $$\begin{aligned}&\sum _{k=1}^i\tau _n(G^{-1}_n(eu_n^k+e\delta u_n^k),e u_n^k)_{p,p'}\nonumber \\&\quad =\sum _{k=1}^i\tau _n(G^{-1}_n(eu_n^k+e\delta u_n^k),e z_n^k)_{p,p'}+\sum _{k=1}^i\tau _n(f_n^k, u_n^k- z_n^k)_H\nonumber \\&\qquad -\sum _{k=1}^i\tau _n(\delta ^2u_n^k, u_n^k- z_n^k)_H. \end{aligned}$$

Now we use $\varphi =\tau _n(\delta u_n^k-\delta z_n^k)\in V_n^k$ as a test function in (3.6) and we get

3.25 $$\begin{aligned}&\Vert \delta u_n^k\Vert _H^2-(\delta u_n^{k-1},\delta u_n^k)_H+\tau _n(G^{-1}_n(eu_n^k+e\delta u_n^k), e \delta u_n^k)_{p,p'}\nonumber \\&\quad =\tau _n( f_n^k, \delta u_n^k-\delta z_n^k)_H+\tau _n(G^{-1}_n(eu_n^k+e\delta u_n^k),e \delta z_n^k)_{p,p'}+\tau _n (\delta ^2 u_n^k, \delta z_n^k)_H. \end{aligned}$$

By means of the following identity

$$\begin{aligned} \Vert \delta u_n^k\Vert _H^2-(\delta u_n^{k-1},\delta u_n^k)_H=\frac{1}{2}\Vert \delta u_n^k\Vert _H^2-\frac{1}{2}\Vert \delta u_n^{k-1}\Vert _H^2+\frac{\tau _n^2}{2}\Vert \delta ^2 u_n^k\Vert _H^2, \end{aligned}$$

from (3.25) we infer

$$\begin{aligned}&\frac{1}{2}\Vert \delta u_n^k\Vert _H^2-\frac{1}{2}\Vert \delta u_n^{k-1}\Vert _H^2+\frac{\tau _n^2}{2}\Vert \delta ^2 u_n^k\Vert _H^2+\tau _n(G^{-1}_n(eu_n^k+e\delta u_n^k),e \delta u_n^k)_{p,p'}\\&\quad =\tau _n( f_n^k, \delta u_n^k-\delta z_n^k)_H+\tau _n(G^{-1}_n(eu_n^k+e\delta u_n^k),e \delta z_n^k)_{p,p'}+\tau _n (\delta ^2 u_n^k, \delta z_n^k)_H, \end{aligned}$$

and, by summing again over $k\in \{1,\dots ,i\}$ we get

3.26 $$\begin{aligned}&\frac{1}{2}\Vert \delta u_n^i\Vert _H^2-\frac{1}{2}\Vert u^1\Vert _H^2+\sum _{k=1}^i\tau _n(G^{-1}_n(eu_n^k+e\delta u_n^k),e \delta u_n^k)_{p,p'}\nonumber \\&\quad \le \sum _{k=1}^i\tau _n(f_n^k, \delta u_n^k-\delta z_n^k)_H+\sum _{k=1}^i\tau _n(G^{-1}_n(eu_n^k+e\delta u_n^k),e \delta z_n^k)_{p,p'}\nonumber \\&\qquad +\sum _{k=1}^i\tau _n (\delta ^2 u_n^k, \delta z_n^k)_H. \end{aligned}$$

By considering together (3.24) and (3.26) we get

$$\begin{aligned}&\frac{1}{2}\Vert \delta u_n^i\Vert _H^2+\sum _{k=1}^i\tau _n(G^{-1}_n(eu_n^k+e\delta u_n^k),eu_n^k+e \delta u_n^k)_{p,p'}\\&\quad \le \frac{1}{2}\Vert u^1\Vert _H^2+\sum _{k=1}^i\tau _n(G^{-1}_n(eu_n^k+e\delta u_n^k),ez_n^k+e \delta z_n^k)_{p,p'}\\&\qquad +\sum _{k=1}^i\tau _n(f_n^k, u_n^k+\delta u_n^k- z_n^k-\delta z_n^k)_H+\sum _{k=1}^i\tau _n (\delta ^2 u_n^k, z_n^k+ \delta z_n^k)_H\\&\qquad -\sum _{k=1}^i\tau _n (\delta ^2 u_n^k, u_n^k)_H. \end{aligned}$$

Thanks to (3.1)–(3.3) and the Korn-Poincaré inequality (2.3) we deduce from the previous estimate

3.27 $$\begin{aligned}&\frac{1}{2}\Vert \delta u_n^i\Vert _H^2+\frac{c_1}{C_{KP}^{p'}}\sum _{k=1}^i\tau _n\Vert u_n^k+\delta u_n^k\Vert ^{p'}_V \nonumber \\ {}&\quad \le c_2T{\mathcal {L}}^d(\Omega )+\frac{1}{2}\Vert u^1\Vert _H^2+\sum _{k=1}^i\tau _n(G^{-1}_n(eu_n^k+e\delta u_n^k),ez_n^k+e \delta z_n^k)_{p,p'}\nonumber \\ {}&\qquad +\sum _{k=1}^i\tau _n(f_n^k, u_n^k+\delta u_n^k- z_n^k-\delta z_n^k)_H+\sum _{k=1}^i\tau _n (\delta ^2 u_n^k, z_n^k+\delta z_n^k)_H\nonumber \\ {}&\qquad -\sum _{k=1}^i\tau _n (\delta ^2 u_n^k, u_n^k)_H. \end{aligned}$$

Let us now estimate the right-hand side of (3.27) from above. We can write

3.28 $$\begin{aligned}&\left| \sum _{k=1}^i \tau _n(f_n^k, u_n^k+\delta u_n^k)_H\right| \le \Vert f\Vert ^2_{L^2(0,T;H)}+\frac{1}{2}\sum _{k=1}^i \tau _n\Vert u_n^k\Vert _H^2+\frac{1}{2}\sum _{k=1}^i \tau _n\Vert \delta u_n^k\Vert _H^2,\end{aligned}$$

3.29 $$\begin{aligned}&\left| \sum _{k=1}^i \tau _n( f_n^k, z_n^k+\delta z_n^k)_H\right| \le \Vert f\Vert ^2_{L^2(0,T;H)}+\frac{T}{2}\Vert z\Vert _{L^\infty (0,T;H)}^2+\frac{1}{2}\Vert \dot{z}\Vert _{L^2(0,T;H)}^2. \end{aligned}$$

Moreover

3.30 $$\begin{aligned}&\left| \sum _{k=1}^i\tau _n(G^{-1}_n(eu_n^k+e\delta u_n^k),ez_n^k+e \delta z_n^k)_{p,p'}\right| \nonumber \\&\quad \le \frac{\varepsilon ^p}{p}\sum _{k=1}^i\tau _n\Vert G^{-1}_n(eu_n^k+e\delta u_n^k)\Vert _p^p+\frac{1}{p'\varepsilon ^{p'}}\sum _{k=1}^i\tau _n\Vert ez_n^k+e\delta z_n^k\Vert _{p'}^{p'}\nonumber \\&\quad \le \frac{2^{p-1}c^p_3\varepsilon ^p}{p}\sum _{k=1}^i\tau _n\Vert u_n^k+\delta u_n^k\Vert _V^{p'}+\frac{2^{p-1}c^p_3T \varepsilon ^p}{p}{\mathcal {L}}^d(\Omega )+\frac{2^{p'-1}T}{p'\varepsilon ^{p'}}\Vert z\Vert _{L^\infty (0,T;V)}^{p'} \nonumber \\&\qquad +\frac{2^{p'-1}}{p'\varepsilon ^{p'}}\Vert \dot{z}\Vert _{L^{p'}(0,T;V)}^{p'}. \end{aligned}$$

Notice that the following discrete integration by parts formulas hold

3.31 $$\begin{aligned}&\sum _{k=1}^i \tau _n(\delta ^2 u_n^k,z_n^k+\delta z^k_n)_H=(\delta u_n^i,z_n^i+\delta z_n^i)_H-(\delta u_n^0,z_n^0+\delta z_n^0)_H \nonumber \\ {}&\quad -\sum _{k=1}^i\tau _n (\delta u_n^{k-1},\delta z_n^k+\delta ^2 z_n^k)_H, \end{aligned}$$

3.32 $$\begin{aligned}&\sum _{k=1}^i \tau _n(\delta ^2 u_n^k,u_n^k)_H=(\delta u_n^i,u_n^i)_H-(\delta u_n^0,u_n^0)_H\nonumber \\ {}&\quad -\sum _{k=1}^i\tau _n (\delta u_n^{k-1},\delta u_n^k)_H. \end{aligned}$$

Since

3.33 $$\begin{aligned} \sum _{k=1}^i \tau _n \Vert \delta u_n^{k-1}\Vert _H^2=\sum _{k=0}^{i-1} \tau _n \Vert \delta u_n^k\Vert _H^2\le T\Vert u^1\Vert _H^2+\sum _{k=1}^i \tau _n \Vert \delta u_n^k\Vert _H^2, \end{aligned}$$

thanks to (3.31) we can write for all $\varepsilon >0$

3.34 $$\begin{aligned}&\Big | \sum _{k=1}^i \tau _n(\delta ^2 u_n^k,z_n^k+\delta z^k_n)_H\Big |\nonumber \\&\quad \le \frac{\varepsilon }{2}\Vert \delta u_n^i\Vert _H^2+\frac{1}{2\varepsilon }\Vert z_n^i+\delta z_n^i\Vert _H^2+\Vert u^1\Vert _H\Vert z(0)+{\dot{z}}(0)\Vert _H\nonumber \\&\qquad +\frac{1}{2} \sum _{k=1}^i \tau _n \Vert \delta u_n^{k-1}\Vert _H^2+\frac{1}{2} \sum _{k=1}^i \tau _n \Vert \delta z_n^k+\delta ^2 z_n^k\Vert _H^2\nonumber \\&\quad \le K_1^\varepsilon +\frac{\varepsilon }{2}\Vert \delta u_n^i\Vert _H^2+\frac{1}{2}\sum _{k=1}^i \tau _n \Vert \delta u_n^k\Vert _H^2, \end{aligned}$$

where $K_1^\varepsilon$ is a positive constant depending on $\varepsilon$. Moreover, since $u_n^i=\sum _{k=1}^i\tau _n\delta u_n^k+u^0$ for all $i\in \{1,\dots ,n\}$, the discrete Hölder inequality gives us

3.35 $$\begin{aligned} \Vert u_n^i\Vert _H\le \sum _{k=1}^i\tau _n\Vert \delta u_n^k\Vert _H+\Vert u^0\Vert _H\le T^{\frac{1}{2}}\left( \sum _{k=1}^i\tau _n\Vert \delta u_n^k\Vert _H^2\right) ^{\frac{1}{2}}+\Vert u^0\Vert _H. \end{aligned}$$

Hence from (3.32), (3.33), and (3.35) we deduce

3.36 $$\begin{aligned} \left| \sum _{k=1}^i \tau _n(\delta ^2 u_n^k,u_n^k)_H\right|&\le \frac{\varepsilon }{2}\Vert \delta u_n^i\Vert _H^2+\frac{1}{2\varepsilon }\Vert u_n^i\Vert _H^2+\Vert u^1\Vert _H\Vert u^0\Vert _H \nonumber \\&\quad +\frac{1}{2} \sum _{k=1}^i \tau _n \Vert \delta u_n^{k-1}\Vert _H^2+\frac{1}{2} \sum _{k=1}^i \tau _n \Vert \delta u_n^k\Vert _H^2\nonumber \\&\le K_2^\varepsilon +\frac{\varepsilon }{2}\Vert \delta u_n^i\Vert _H^2+\left( 1+\frac{T}{\varepsilon }\right) \sum _{k=1}^i \tau _n \Vert \delta u_n^k\Vert _H^2, \end{aligned}$$

where $K_2^\varepsilon$ is a positive constant depending on $\varepsilon$. Furthermore

3.37 $$\begin{aligned} \frac{1}{2}\sum _{k=1}^i \tau _n\Vert u_n^k\Vert _H^2\le T\Vert u^0\Vert _H^2+T^2\sum _{k=1}^i\tau _n\Vert \delta u_n^i\Vert _H^2. \end{aligned}$$

If we consider together (3.27)–(3.37), we get

$$\begin{aligned}&\Big (\frac{1}{2}-\varepsilon \Big )\Vert \delta u_n^i\Vert _H^2+\left( \frac{c_1}{C_{KP}^{p'}}-\frac{2^{p-1}c^p_3\varepsilon ^p}{p}\right) \sum _{k=1}^i\tau _n\Vert u_n^k+\delta u_n^k\Vert ^{p'}_{V}\\&\quad \le K_3^\varepsilon \left( 1+\sum _{k=1}^i\tau _n\Vert \delta u_n^k\Vert _H^2\right) , \end{aligned}$$

where $K_3^\varepsilon$ is a positive constant depending on $\varepsilon$. By choosing

$$\begin{aligned} 0<\varepsilon <\min \left\{ \frac{1}{2},\Big (\frac{c_1 p}{C_{KP}^{p'}2^{p-1}c^p_3}\Big )^{\frac{1}{p}}\right\} \end{aligned}$$

we get the existence of a positive constant $K_4$ independent of *n* and *i* such that

3.38 $$\begin{aligned} \Vert \delta u_n^i\Vert _H^2+\sum _{k=1}^i\tau _n\Vert u_n^k+\delta u_n^k\Vert ^{p'}_{V}\le K_4\left( 1+\sum _{k=1}^i\tau _n\Vert \delta u_n^k\Vert _H^2\right) . \end{aligned}$$

By defining $a_n^i:=\Vert \delta u_n^i\Vert _H^2$ for all $i\in \{1,\dots ,n\}$, from (3.38) we derive

$$\begin{aligned} a_n^i\le K_4\left( 1+\sum _{k=1}^i \tau _n a_n^k\right) \quad \text {for all}\ i\in \{1,\dots ,n\}, \end{aligned}$$

and taking into account a discrete version of Gronwall lemma (see, e.g., [1, Lemma 3.2.4]) we deduce that the family $\{a_n^i\}_{i=1}^n$ is bounded by a positive constant $K_5$ independent of *i* and *n*, i.e.,

3.39 $$\begin{aligned} \Vert \delta u_n^i\Vert _H^2\le K_5\quad \text{ for } \text{ all }\ i\in \{1,\dots ,n\}\ \text{ and }\ n\in {\mathbb {N}}. \end{aligned}$$

By using (3.38) and (3.39) we get the existence of a positive constant $K_6$ independent of *n* such that

3.40 $$\begin{aligned} \max _{i\in \{1,\dots ,n\}}\Vert \delta u_n^i\Vert _H^2+\sum _{i=1}^n \tau _n \Vert u_n^i+\delta u_n^i\Vert ^{p'}_{V}\le K_6. \end{aligned}$$

In particular, by (3.3) and (3.21) it holds

3.41 $$\begin{aligned} \sum _{i=1}^n\tau _n\Vert \sigma _n^i\Vert _p^p\le & {} 2^{p-1}c_3^p\sum _{i=1}^n\tau _n\Vert eu_n^i+e\delta u_n^i\Vert _{p'}^{p'}+2^{p-1}c_3^p T{\mathcal {L}}^d(\Omega ) \nonumber \\ {}\le & {} 2^{p-1}c_3^pK_6+2^{p-1}c_3^p T{\mathcal {L}}^d(\Omega ). \end{aligned}$$

To get the last estimate in (3.22) we set $b_n^k:=(1+\tau _n)^k$ for $k\in \{0,\dots ,n\}$ and we notice that

3.42 $$\begin{aligned} \frac{b_n^k-b_n^{k-1}}{\tau _n}=b_n^{k-1}\quad \text {for all}\ k\in \{1,\dots ,n\}. \end{aligned}$$

From (3.42) we can write

3.43 $$\begin{aligned} b_n^ku_n^k-b_n^0u_n^0&=\sum _{j=1}^k(b_n^ju_n^j-b_n^{j-1}u_n^{j-1})\nonumber \\ {}&=\sum _{j=1}^k\tau _n \frac{b_n^j-b_n^{j-1}}{\tau _n}u_n^j+\sum _{j=1}^k\tau _n b_n^{j-1} \frac{u_n^j-u_n^{j-1}}{\tau _n}=\sum _{j=1}^k\tau _n b_n^{j-1}(u_n^j+\delta u_n^j). \end{aligned}$$

Since

$$\begin{aligned} 1\le (1+\tau _n)^k\le (1+\tau _n)^n=\left[ \left( 1+\frac{T}{n}\right) ^{\frac{n}{T}}\right] ^T\le \mathrm e^T, \end{aligned}$$

from (3.43) we deduce the existence of a positive constant $K_7$ such that

3.44 $$\begin{aligned} \Vert u_n^k\Vert ^{p'}_{V}\le K_7\left( 1+\sum _{j=1}^k\tau _n \Vert u_n^j+\delta u_n^j\Vert ^{p'}_{V}\right) \le K_7\left( 1+K_6\right) . \end{aligned}$$

As a consequence of this, we obtain

3.45 $$\begin{aligned} \sum _{j=1}^k\tau _n \Vert \delta u_n^j\Vert ^{p'}_{V}\le & {} 2^{p'-1}\sum _{j=1}^k\tau _n (\Vert u_n^j+\delta u_n^j\Vert ^{p'}_{V}+\Vert u_n^j\Vert ^{p'}_{V})\nonumber \\\le & {} 2^{p'-1}\left( K_6+TK_7+TK_7K_6\right) . \end{aligned}$$

Hence by considering together (3.40), (3.41), (3.44), and (3.45) we get (3.22). $\square$

As a consequence of (3.22) and of the particular form of equation (3.6), we derive also a uniform bound on the discrete second time derivative of $u_n^k$ in the space *H*. This allows us to find in the limit as $n\rightarrow \infty$ a weak solution to (2.6)–(2.8) with displacement $u\in W^{2,2}(0,T;H)$.

### Corollary 3.4

There exists a constant $C_2$, independent of $n\in {\mathbb {N}}$, such that

3.46 $$\begin{aligned} \sum _{k=1}^n\tau _n\Vert \delta ^2 u^k_n\Vert _H^2\le C_2. \end{aligned}$$

### Proof

Let us define $v_n^k:=u_n^k+\delta u_n^k\in V$ for all $k\in \{1,\dots ,n\}$ and $n\in {\mathbb {N}}$. By equation (3.6) we deduce that $v_n^k$ solves the following equation

$$\begin{aligned} (\delta v_n^k,\varphi )_H+(G^{-1}_n(ev_n^k),e\varphi )_{p,p'}=(f_n^k+\delta u_n^k,\varphi )_H\quad \text {for all}\ \varphi \in V_n^k. \end{aligned}$$

We take $\varphi :=\tau _n(\delta v_n^k-\delta z_n^k-\delta ^2 z_n^k)\in V_n^k$ as a test function in (3.6). We fix $i\in \{1,\dots ,n\}$ and by summing over $k\in \left\{ 1,\dots i\right\}$ we get

3.47 $$\begin{aligned}&\sum _{k=1}^i\tau _n\Vert \delta v_n^k\Vert _H^2+\sum _{k=1}^i(G^{-1}_n(ev_n^k),e v_n^k-ev_n^{k-1})_{p,p'}\nonumber \\&\quad =\sum _{k=1}^i\tau _n(f_n^k+\delta u_n^k,\delta v_n^k)_H-\sum _{k=1}^i\tau _n(f_n^k+\delta u_n^k,\delta z_n^k+\delta ^2 z_n^k)_H\nonumber \\&\qquad +\sum _{k=1}^i\tau _n (\delta v_n^k,\delta z_n^k+\delta ^2 z_n^k)_H+\sum _{k=1}^i\tau _n(G^{-1}_n(ev_n^k),e\delta z_n^k+e\delta ^2 z_n^k)_{p,p'}. \end{aligned}$$

Let us now estimate the right-hand side of (3.47) from above. Thanks to (3.22) we can write

3.48 $$\begin{aligned}&\left| \sum _{k=1}^i \tau _n(f_n^k+\delta u_n^k, \delta v_n^k)_H\right| \le \frac{1}{2\varepsilon }\Vert f\Vert ^2_{L^2(0,T;H)}+\frac{T C_1^2}{2\varepsilon }+\varepsilon \sum _{k=1}^i \tau _n\Vert \delta v_n^k\Vert ^2_H,\end{aligned}$$

3.49 $$\begin{aligned}&\left| \sum _{k=1}^i \tau _n(f_n^k+\delta u_n^k, \delta z_n^k+\delta ^2 z_n^k)_H\right| \le \Vert f\Vert ^2_{L^2(0,T;H)}+TC_1^2+\Vert {\dot{z}}\Vert ^2_{W^{1,2}(0,T;H)},\end{aligned}$$

3.50 $$\begin{aligned}&\left| \sum _{k=1}^i\tau _n (\delta v_n^k,\delta z_n^k+\delta ^2 z_n^k)_H\right| \le \varepsilon \sum _{k=1}^i \tau _n\Vert \delta v_n^k\Vert ^{2}_{H}+\frac{1}{2\varepsilon }\Vert {\dot{z}}\Vert _{W^{1,2}(0,T;H)}^2. \end{aligned}$$

Moreover

3.51 $$\begin{aligned}&\left| \sum _{k=1}^i\tau _n(G^{-1}_n(ev_n^k),e\delta z_n^k+e \delta ^2 z_n^k)_{p,p'}\right| \nonumber \\&\quad \le \frac{1}{p}\sum _{k=1}^i\tau _n\Vert G^{-1}_n(ev_n^k)\Vert _p^p+\frac{1}{p'}\sum _{k=1}^i\tau _n\Vert e\delta z_n^k+e\delta ^2 z_n^k\Vert _{p'}^{p'}\nonumber \\&\quad \le \frac{2^{p-1}c_3^p}{p}\sum _{k=1}^i\tau _n\Vert u_n^k+\delta u_n^k\Vert _V^{p'}+\frac{2^{p-1}c_3^pT}{p}{\mathcal {L}}^d(\Omega )+\frac{2^{p-1}}{p'}\Vert \dot{z}\Vert _{W^{1,p'}(0;T;V)}^{p'}\nonumber \\&\quad \le \frac{4^{p-1}c_3^p}{p}(TC_1^p+C_1)+\frac{2^{p-1}c_3^pT}{p}{\mathcal {L}}^d(\Omega )+\frac{2^{p-1}}{p'}\Vert {\dot{z}}\Vert _{W^{1,p'}(0;T;V)}^{p'}. \end{aligned}$$

Finally, by (3.1) and the convexity of $\phi _n^*$ we have

3.52 $$\begin{aligned} \sum _{k=1}^i(G^{-1}_n(ev_n^k),e v_n^k-ev_n^{k-1})_{p,p'}&\ge \sum _{k=1}^i \left( \int _\Omega \phi _n^*(ev_n^k(x))\,\mathrm dx-\int _\Omega \phi _n^*(ev_n^{k-1}(x))\,\mathrm dx\right) \nonumber \\&=\int _\Omega \phi _n^*(ev_n^i(x))\,\mathrm dx-\int _\Omega \phi _n^*(ev_n^0(x))\,\mathrm dx. \end{aligned}$$

By combining (3.47)–(3.52) with the bound (3.5) for $\phi _n^*$, we deduce the existence of a positive constant $K^\varepsilon$, which depends on $\varepsilon$, but it is independent of *n* and *i*, such that

3.53 $$\begin{aligned} (1-2\varepsilon )\sum _{k=1}^i\tau _n\Vert \delta v_n^k\Vert ^2_H+\int _\Omega \phi _n^*(ev_n^i(x))\,\mathrm dx\le K^\varepsilon \quad \text {for all}\ i\in \{1,\dots ,n\}.\nonumber \\ \end{aligned}$$

By choosing $\varepsilon =\frac{1}{4}$ and using (3.4), from (3.22) and (3.53) we deduce (3.46). $\square$

We now want to pass to the limit as $n\rightarrow \infty$ in the discrete equation (3.6) to obtain a weak solution $(u,\sigma )$ to the nonlinear viscoelastic system (2.6)–(2.8), according to Definition 2.3. We start by defining the following interpolation sequences of $\{(u_n^k,\sigma _n^k)\}_{k=1}^n$:

$$\begin{aligned} \begin{array}{lll} u_n(t):=u_n^k+(t-k\tau _n)\delta u_n^k, &{}{\tilde{u}}_n(t):=\delta u_n^k+(t-k\tau _n)\delta ^2 u_n^k, &{} t\in [(k-1)\tau _n,k\tau _n],\quad k\in \{1,\dots ,n\},\\ u^+_n(t):=u_n^k, &{}{\tilde{u}}^+_n(t):=\delta u_n^k, &{} t\in ((k-1)\tau _n,k\tau _n], \quad k\in \{1,\dots ,n\},\\ u^+_n(0):=u_n^0=u^0, &{}{\tilde{u}}^+_n(t):=\delta u_n^0=u^1, &{}\\ u^-_n(t):=u_n^{k-1}, &{}{\tilde{u}}^-_n(t):=\delta u_n^{k-1},&{} t\in [(k-1)\tau _n,k\tau _n), \quad k\in \{1,\dots ,n\},\\ u^-_n(T):=u_n^n, &{}{\tilde{u}}^-_n(T):=\delta u_n^n,&{} \\ \sigma _n^+(t):=\sigma _n^k,&{} &{} t\in ((k-1)\tau _n,k\tau _n], \quad k\in \{1,\dots ,n\}. \end{array} \end{aligned}$$

By means of this notation, we can state the following convergence lemma.

### Lemma 3.5

There exists a pair $(u,\sigma )\in ({\mathcal {V}}\cap W^{2,2}(0,T;H))\times L^p(0,T;L^p(\Omega ;{\mathbb {R}}^{d\times d}_{sym}))$ such that, up to a not relabeled subsequence3.543.55Moreover3.563.57

### Proof

Thanks to Lemma 3.3 and the estimate (3.46), the sequences

$$\begin{aligned}&\{u_n\}_n\subset W^{1,p'}(0, T;V)\cap W^{1,\infty }(0, T;H),{} & {} \{{{\tilde{u}}}_n\}_n\subset L^{p'}(0,T;V)\cap W^{1,2}(0,T;H),\\&\{\sigma _n^+\}_n\subset L^p(0,T;L^p(\Omega ;{\mathbb {R}}^{d\times d}_{sym})), \end{aligned}$$

are uniformly bounded with respect to $n\in {\mathbb {N}}$. Indeed, we have

$$\begin{aligned}&\Vert u_n\Vert ^{p'}_{W^{1,p'}(0, T;V)}\le T\max _{k\in \{0,\dots ,n\}}\Vert u_n^k\Vert _V^{p'}+\sum _{k=1}^n\tau _n\Vert \delta u_n^k\Vert ^{p'}_V,\\&\Vert u_n\Vert _{W^{1,\infty }(0, T;H)}\le \max _{k\in \{0,\dots ,n\}}\Vert u_n^k\Vert _H+\max _{k\in \{1,\dots ,n\}}\Vert \delta u_n^k\Vert _H,\\&\Vert {\tilde{u}}_n\Vert _{L^{p'}(0,T;V)}^{p'}\le 2\sum _{k=1}^n\tau _n\Vert \delta u_n^k\Vert _V^{p'}+\Vert u^1\Vert _V^{p'},\\&\Vert {\tilde{u}}_n\Vert _{W^{1,2}(0,T;H)}^2\le T\max _{k\in \{0,\dots ,n\}}\Vert \delta u_n^k\Vert _H^2+\sum _{k=1}^n\tau _n\Vert \delta ^2 u_n^k\Vert ^2_H,\\&\Vert \sigma _n^+\Vert ^p_{L^p(0,T;L^p(\Omega ;{\mathbb {R}}^{d\times d}_{sym}))}= \sum _{k=1}^n\tau _n\Vert \sigma _n^k\Vert _p^p. \end{aligned}$$

By Banach-Alaoglu theorem and Lemma 2.5 there exist three functions $u\in W^{1,p'}(0, T;V)\cap W^{1,\infty }(0, T;H)$, $v\in L^{p'}(0,T;V)\cap W^{1,2}(0,T;H)$, and $\sigma \in L^p(0,T;L^p(\Omega ;{\mathbb {R}}^{d\times d}_{sym}))$ such that, up to a not relabeled subsequence3.58andThanks to (3.46) we get

$$\begin{aligned} \Vert \dot{u}_n-{{\tilde{u}}}_n\Vert _{L^2(0,T;H)}^2\le \tau _n^2\sum _{k=1}^n\tau _n\Vert \delta ^2 u_n^k\Vert ^2_H\le C_2\tau _n^2\xrightarrow [n\rightarrow \infty ]{}0, \end{aligned}$$

from which we deduce that $v=\dot{u}$.

By (3.22) also the sequences

3.59 $$\begin{aligned}&\{u_n^\pm \}_n\subset L^\infty (0, T;V),{} & {} \{\tilde{u}_n^\pm \}_n\subset L^{p'}(0,T;V)\cap L^\infty (0,T;H), \end{aligned}$$

are uniformly bounded. Moreover, by using again (3.22) and (3.46) we have

$$\begin{aligned}&\Vert u_n-u^{+}_n\Vert _{L^\infty (0, T;H)} \le \tau _n\max _{k\in \{1,\dots ,n\}}\ \Vert \delta u_n^k\Vert _H\le C_1\tau _n\xrightarrow [n\rightarrow \infty ]{}0,\\&\Vert u^+_n-u^{-}_n\Vert _{L^\infty (0, T;H)}\le \tau _n \max _{k\in \{1,\dots ,n\}}\ \Vert \delta u_n^k\Vert _H\le C_1\tau _n\xrightarrow [n\rightarrow \infty ]{}0,\\&\Vert {{\tilde{u}}}_n-{{\tilde{u}}}_n^+\Vert _{L^2(0,T;H)}^2\le \tau _n^2\sum _{k=1}^n\tau _n\Vert \delta ^2 u_n^k\Vert ^2_{H}\le C_2 \tau _n^2\xrightarrow [n\rightarrow \infty ]{}0,\\&\Vert {{\tilde{u}}}^+_n-{{\tilde{u}}}_n^-\Vert _{L^2(0,T;H)}^2\le \tau _n^2\sum _{k=1}^n\tau _n\Vert \delta ^2 u_n^k\Vert ^2_H\le C_2 \tau _n^2\xrightarrow [n\rightarrow \infty ]{}0. \end{aligned}$$

We combine (3.58) and (3.59) with the previous convergences to deriveFinally, by (3.58) for all $t\in [0,T]$ we haveThanks to (3.22) and (3.46), for all $t\in [0,T]$ we get

$$\begin{aligned} \begin{aligned} \Vert u_n^\pm (t)\Vert _{V}\le C_1,\quad \Vert u_n^+(t)-u_n(t)\Vert _H&\le C_1\tau _n\xrightarrow [n\rightarrow \infty ]{}0,\\ \Vert u_n^+(t)-u^-_n(t)\Vert _H&\le C_1\tau _n\xrightarrow [n\rightarrow \infty ]{}0,\\ \Vert {{\tilde{u}}}_n^\pm (t)\Vert _H\le C_1,\quad \Vert {{\tilde{u}}}_n^+(t)-{{\tilde{u}}}_n(t)\Vert _{H}^2&\le \tau _n\sum _{k=1}^n\tau _n\Vert \delta ^2 u_n^k\Vert ^2_H\le C_2\tau _n\xrightarrow [n\rightarrow \infty ]{}0,\\ \Vert {{\tilde{u}}}_n^+(t)-\tilde{u}^-_n(t)\Vert _H^2&=\tau _n\sum _{k=1}^n\tau _n\Vert \delta ^2 u_n^k\Vert _H^2\le C_2\tau _n\xrightarrow [n\rightarrow \infty ]{}0, \end{aligned} \end{aligned}$$

which imply (3.56) and (3.57). $\square$

In view of the compactness of the embedding $V\hookrightarrow H$ (see Remark 2.1), we deduce also the following strong convergences.

### Corollary 3.6

Let $(u,\sigma )\in ({\mathcal {V}}\cap W^{2,2}(0,T;H))\times L^p(0,T;L^p(\Omega ;{\mathbb {R}}^{d\times d}_{sym}))$ be the pair of functions given by Lemma 3.5. Then, we have

3.60 $$\begin{aligned} u_n^+\xrightarrow [n\rightarrow \infty ]{L^2(0,T;H)} u,\quad \tilde{u}_n^+\xrightarrow [n\rightarrow \infty ]{L^2(0,T;H)} \dot{u}. \end{aligned}$$

### Proof

By Lemma 3.5 we know that the following sequences

$$\begin{aligned}&\{u_n\}_n\subset W^{1,p'}(0, T;V)\cap W^{1,\infty }(0, T;H),{} & {} \{{{\tilde{u}}}_n\}_n\subset L^{p'}(0,T;V)\cap W^{1,2}(0,T;H), \end{aligned}$$

are uniformly bounded with respect to *n*. Since the embedding $V\hookrightarrow H$ is compact, by Aubin-Lions lemma (see for example [24, Theorem 3]), we derive

$$\begin{aligned} u_n\xrightarrow [n\rightarrow \infty ]{L^2(0,T;H)} u,\quad \tilde{u}_n\xrightarrow [n\rightarrow \infty ]{L^2(0,T;H)} \dot{u}. \end{aligned}$$

Moreover, we have

$$\begin{aligned}&\Vert u_n-u^+_n\Vert _{L^2(0, T;H)}^2 \le \tau _n^2\sum _{k=1}^n\tau _n\Vert \delta u_n^k\Vert ^2_H\le TC_1\tau _n^2\xrightarrow [n\rightarrow \infty ]{}0,\\&\Vert {{\tilde{u}}}_n-{{\tilde{u}}}_n^+\Vert _{L^2(0,T;H)}^2\le \tau _n^2\sum _{k=1}^n\tau _n\Vert \delta ^2 u_n^k\Vert ^2_{H}\le C_2 \tau _n^2\xrightarrow [n\rightarrow \infty ]{}0, \end{aligned}$$

which imply (3.60). $$\square $$

We want to prove that the pair $(u,\sigma )\in ({\mathcal {V}}\cap W^{2,2}(0,T;H))\times L^p(0,T;L^p(\Omega ;{\mathbb {R}}^{d\times d}_{sym}))$ of Lemma 3.5 is a weak solution to the nonlinear viscoelastic system (2.6)–(2.8) with initial conditions (2.9). To this aim, we need to check (*i*)–(*iii*) of Definition 2.3 and that $u(0)=u^0$ in *V* and $\dot{u}(0)=u^1$ in *H*. We start by showing that the function $u\in {\mathcal {V}}\cap W^{2,2}(0,T;H)$ satisfies the Dirichlet boundary conditions and the initial conditions.

### Lemma 3.7

The function $u\in {\mathcal {V}}\cap W^{2,2}(0,T;H)$ of Lemma 3.5 satisfies (*i*) of Definition 2.3 and the initial conditions $u(0)=u^0$ in *V* and $\dot{u}(0)=u^1$ in *H*.

### Proof

By (3.56) we haveHence, $u(0)=u^0$ in *V* and $\dot{u}(0)=u^1$ in *H*. Moreover, since $z\in C^0([0,T];V_0)$ and thanks to (3.57), we have for all $t\in [0,T]$Thus, $u(t)-z(t)\in V_t^D$ for all $t\in [0,T]$, being $V_t^D$ a closed subspace of *V*. $\square$

With the next lemma, we show that the pair $(u,\sigma )$ solves the weak formulation (2.10) of the elastodynamics system.

### Lemma 3.8

The pair $(u,\sigma )\in ({\mathcal {V}}\cap W^{2,2}(0,T;H))\times L^{p}(0,T;L^p(\Omega ;{\mathbb {R}}^{d\times d}_{sym}))$ of Lemma 3.5 satisfies (*ii*) of Definition 2.3.

### Proof

We fix $n\in {\mathbb {N}}$ and a function $\varphi \in {\mathcal {D}}$. We consider the following functions

$$\begin{aligned} \begin{aligned} \varphi _n^k&:=\varphi (k\tau _n)\quad \text {for}\ k\in \{0,\dots ,n\},\quad \delta \varphi _n^k&:=\frac{\varphi _n^k-\varphi _n^{k-1}}{\tau _n}\quad \text {for }k\in \{1,\dots ,n\}, \end{aligned} \end{aligned}$$

and the piecewise-constant approximating sequences

$$\begin{aligned}&\varphi ^+_n(t):=\varphi _n^k,{} & {} {{\tilde{\varphi }}}^+_n(t):=\delta \varphi _n^k,{} & {} f^+_n(t):=f_n^k,{} & {} \text {for } t\in ((k-1)\tau _n,k\tau _n],\quad k\in \{1,\dots ,n\}. \end{aligned}$$

If we use $\tau _n\varphi _n^k\in V_n^k$ as a test function in (3.6), after summing over $k\in \{1,\dots ,n\}$, we get

3.61 $$\begin{aligned} \sum _{k=1}^n\tau _n(\delta ^2u_n^k,\varphi ^k_n)_H+\sum _{k=1}^n\tau _n(\sigma _n^k,e\varphi ^k_n)_{p,p'} =\sum _{k=1}^n\tau _n(f_n^k,\varphi ^k_n)_H. \end{aligned}$$

Since $\varphi _n^0=\varphi _n^n=0$ we obtain

$$\begin{aligned}&\sum _{k=1}^n \tau _n(\delta ^2 u^k_n,\varphi ^k_n)_H \\&\quad =\sum _{k=1}^{n} (\delta u^k_n,\varphi ^k_n)_H-\sum _{k=1}^n (\delta u^{k-1}_n,\varphi ^k_n)_H =\sum _{k=0}^{n-1} (\delta u^k_n,\varphi ^k_n)_H-\sum _{k=0}^{n-1} (\delta u^k_n,\varphi ^{k+1}_n)_H\\&\quad =-\sum _{k=0}^{n-1} \tau _n (\delta u^k_n,\delta \varphi ^{k+1}_n)_H=-\sum _{k=1}^n\tau _n(\delta u^{k-1}_n,\delta \varphi ^k_n)_H=-\int _0^T({\tilde{u}}^-_n(t),{\tilde{\varphi }}^+_n(t))_H\, \mathrm dt, \end{aligned}$$

and from (3.61) we deduce

3.62 $$\begin{aligned} -\int _0^T({\tilde{u}}^-_n(t),{\tilde{\varphi }}^+_n(t))_H\,\mathrm dt+\int _0^T(\sigma _n^+(t),e \varphi ^+_n(t))_{p,p'}\,\mathrm dt=\int _0^T( f^+_n(t),\varphi ^+_n(t))_H\,\mathrm dt. \end{aligned}$$

Thanks to (3.55) and the convergences

$$\begin{aligned} \varphi ^+_n\xrightarrow [n\rightarrow \infty ]{L^{p'}(0,T;V)}\varphi , \quad \varphi ^+_n\xrightarrow [n\rightarrow \infty ]{ L^2(0,T;H)}\varphi , \quad {\tilde{\varphi }}^+_n\xrightarrow [n\rightarrow \infty ]{L^2(0,T;H)}{\dot{\varphi }},\quad f^+_n\xrightarrow [n\rightarrow \infty ]{L^2(0,T;H)}f \end{aligned}$$

we can pass to the limit in (3.62), and we get that the pair $(u,\sigma )\in {\mathcal {V}}\times L^p(0,T;L^p(\Omega ;{\mathbb {R}}^{d\times d}_{sym}))$ satisfies (*ii*) of Definition 2.3. $\square$

Finally, we have that the pair $(u,\sigma )$ satisfies the constitutive law (2.11).

### Lemma 3.9

The pair $(u,\sigma )\in ({\mathcal {V}}\cap W^{2,2}(0,T;H))\times L^{p}(0,T;L^p(\Omega ;{\mathbb {R}}^{d\times d}_{sym}))$ of Lemma 3.5 satisfies (*iii*) of Definition 2.3.

### Proof

In order to verify the constitutive law, we use a modification of Minty method, as done in [3, 20]. Since $\dot{u}\in W^{1,2}(0,T;H)$, by integrating by parts in (2.10) we deduce that $(u,\sigma )$ solve

3.63 $$\begin{aligned}{} & {} \int _0^T(\ddot{u}(t),\varphi (t))_H\,\mathrm dt+\int _0^T(\sigma (t),e \varphi (t))_{p,p'}\,\mathrm dt\nonumber \\{} & {} \quad =\int _0^T(f(t),\varphi (t))_H\,\mathrm dt\quad \text {for all}\ \varphi \in {\mathcal {D}}. \end{aligned}$$

Let us now consider a function $\varphi \in L^{p'}(0,T;V)\cap L^2(0,T;H)$ with $\varphi (t)\in V_t^D$ for a.e. $t\in [0,T]$. Then, there exists a sequence of functions $\{\varphi _n\}_n\subset {\mathcal {D}}$ such that

$$\begin{aligned} \varphi _n\xrightarrow [n\rightarrow \infty ]{L^{p'}(0,T;V)}\varphi ,\quad \varphi _n\xrightarrow [n\rightarrow \infty ]{L^2(0,T;H)}\varphi . \end{aligned}$$

This can be done, for example, by considering a sequence $\{\omega _n\}_n\subset C_c^1((\frac{2}{n},T-\frac{2}{n}))$ with $0\le \omega _n\le 1$ in [0, *T*] for all $n\in {\mathbb {N}}$ and such that $\omega _n(t)\rightarrow 1$ as $n\rightarrow \infty$ for all $t\in (0,T)$, and a sequence $\{\rho _n\}_n \subset C_c^1((0,\frac{1}{n}))$ with $\rho _n\ge 0$ and $\int _{{\mathbb {R}}}\rho _n\,\mathrm dt=1$ for all $n\in {\mathbb {N}}$, and defining

$$\begin{aligned} \varphi _n:=\rho _n*(\omega _n \varphi )\quad \text {for all}\ n\in {\mathbb {N}}\end{aligned}$$

(see also [14, Lemma 2.8]). By testing (3.63) with $\varphi _n$ and passing to the limit as $n\rightarrow \infty$ can deduce that the pair $(u,\sigma )$ satisfies

3.64 $$\begin{aligned} \int _0^T(\ddot{u}(t),\varphi (t))_H\,\mathrm dt+\int _0^T(\sigma (t),e \varphi (t))_{p,p'}\,\mathrm dt=\int _0^T(f(t),\varphi (t))_H\,\mathrm dt \end{aligned}$$

for all $\varphi \in L^{p'}(0,T;V)\cap L^2(0,T;H)$ with $\varphi (t)\in V_t^D$ for a.e. $t\in [0,T]$. Notice that

$$\begin{aligned} \dot{u}-{\dot{z}}\in L^{p'}(0,T;V)\cap L^2(0,T;H),\quad \dot{u}(t)-\dot{z}(t)\in V_t^D\quad \text{ for } \text{ a.e. }\ t\in [0,T], \end{aligned}$$

since $\frac{u(t)-u(t-h)}{h}-\frac{z(t)-z(t-h)}{h}\in V_t^D$ for all $t\in (0,T]$ and $h\in (0,t)$, and

$$\begin{aligned} \frac{u(t)-u(t-h)}{h}-\frac{z(t)-z(t-h)}{h}\rightarrow \dot{u}(t)-{\dot{z}}(t)\quad \text {for a.e.}\ t\in [0,T]\ \text {as}\ h\rightarrow 0. \end{aligned}$$

Hence, by using $\varphi :=u+\dot{u}-z-{\dot{z}}$ in (3.64) we get

3.65 $$\begin{aligned}&\int _0^T(\sigma (t),e u(t)+e\dot{u}(t))_{p,p'}\,\mathrm dt\nonumber \\&\quad =\int _0^T(f(t),u (t)+\dot{u}(t))_H\,\mathrm dt-\int _0^T(\ddot{u}(t),u(t)+\dot{u}(t))_H\,\mathrm dt\nonumber \\&\qquad -\int _0^T(f(t),z(t)+{\dot{z}}(t))_H\,\mathrm dt+\int _0^T(\ddot{u}(t),z(t)+{\dot{z}}(t))_H\,\mathrm dt\nonumber \\&\qquad +\int _0^T(\sigma (t),e z(t)+e{\dot{z}}(t))_{p,p'}\,\mathrm dt. \end{aligned}$$

We now consider equation (3.6) and we use $\varphi =\tau _n(u_n^k+\delta u_n^k-z_n^k-\delta z_n^k)$ as test function. By summing over $k\in \{1,\dots ,n\}$ we get

$$\begin{aligned}&\sum _{k=1}^n\tau _n(G^{-1}_n(eu_n^k+e\delta u_n^k),eu_n^k+e\delta u_n^k)_{p,p'}\\&\quad =\sum _{k=1}^n\tau _n(f_n^k,u_n^k+\delta u_n^k)_H-\sum _{k=1}^n\tau _n(\delta ^2u_n^k,u_n^k+\delta u_n^k)_H\\&\qquad -\sum _{k=1}^n\tau _n(f_n^k,z_n^k+\delta z_n^k)_H+\sum _{k=1}^n\tau _n(\delta ^2u_n^k,z_n^k+\delta z_n^k)_H\\&\qquad +\sum _{k=1}^n\tau _n(G^{-1}_n(eu_n^k+e\delta u_n^k),ez_n^k+e\delta z_n^k)_{p,p'}. \end{aligned}$$

By using the notations introduced before, we can rewrite the previous identity as

3.66 $$\begin{aligned}&\int _0^T(\sigma _n^+(t),G_n(\sigma _n^+(t)))_{p,p'}\,\mathrm dt\nonumber \\&\quad =\int _0^T(G^{-1}_n(eu_n^+(t)+e{{\tilde{u}}}_n^+(t)),eu_n^+(t)+e{{\tilde{u}}}_n^+(t))_{p,p'}\,\mathrm dt\nonumber \\&\quad =\int _0^T(f_n^+(t),u_n^+(t)+{\tilde{u}}_n^+(t))_H\,\mathrm dt-\int _0^T(\dot{{{\tilde{u}}}}_n(t),u_n^+(t)+{{\tilde{u}}}_n^+(t))_H\,\mathrm dt\nonumber \\&\qquad -\int _0^T(f_n^+(t),z_n^+(t)+{{\tilde{z}}}_n^+(t))_H\,\mathrm dt+\int _0^T(\dot{{{\tilde{u}}}}_n(t),z_n^+(t)+{{\tilde{z}}}_n^+(t))_H\,\mathrm dt\nonumber \\&\qquad +\int _0^T(\sigma _n^+(t),ez_n^+(t)+e\tilde{z}_n^+(t))_{p,p'}\,\mathrm dt. \end{aligned}$$

Now we pass to the limit in (3.66) as $n\rightarrow \infty$. Thanks to the strong convergences

$$\begin{aligned}&f_n^+\xrightarrow [n\rightarrow \infty ]{L^2(0,T;H)}f,\quad z_n^+\xrightarrow [n\rightarrow \infty ]{L^{p'}(0,T;V)}z,\quad z_n^+\xrightarrow [n\rightarrow \infty ]{L^2(0,T;H)}z,\\{}&{} \tilde{z}_n^+\xrightarrow [n\rightarrow \infty ]{L^{p'}(0,T;V)}{\dot{z}},\quad \tilde{z}_n^+\xrightarrow [n\rightarrow \infty ]{L^2(0,T;H)}{\dot{z}} \end{aligned}$$

and the convergences in (3.54), (3.55), and (3.60) we deduce that there exists

$$\begin{aligned}&\lim _{n\rightarrow \infty }\int _0^T(\sigma _n^+(t),G_n(\sigma _n^+(t)))_{p,p'}\,\mathrm dt\\&\quad =\int _0^T(f(t),u(t)+\dot{u}(t))_H\,\mathrm dt-\int _0^T(\ddot{u}(t),u(t)+\dot{u}(t))_H\,\mathrm dt\\&\qquad -\int _0^T(f(t),z(t)+{\dot{z}}(t))_H\,\mathrm dt+\int _0^T(\ddot{u}(t),z(t)+{\dot{z}}(t))_H\,\mathrm dt\\&\qquad +\int _0^T(\sigma (t), ez(t)+e{\dot{z}}(t))_{p,p'}\,\mathrm dt\\&\quad =\int _0^T(\sigma (t),e u(t)+e\dot{u}(t))_{p,p'}\,\mathrm dt, \end{aligned}$$

in view of (3.65). Notice that by (3.22)

3.67 $$\begin{aligned}&\Vert G(\sigma _n^+)-eu_n^+-e{{\tilde{u}}}_n^+\Vert _{L^{p'}(0,T;L^{p'}(\Omega ;{\mathbb {R}}^{d\times d}_{sym}))}^{p'}\nonumber \\ {}&\quad =\Vert G(\sigma _n^+)-G_n(\sigma _n^+)\Vert _{L^{p'}(0,T;L^{p'}(\Omega ;{\mathbb {R}}^{d\times d}_{sym}))}^{p'}\nonumber \\ {}&\quad =\frac{1}{n^{p'}}\Vert \sigma _n^+\Vert _{L^{p}(0,T;L^{p}(\Omega ;{\mathbb {R}}^{d\times d}_{sym}))}^p\le \frac{C_1}{n^{p'}}\xrightarrow [n\rightarrow \infty ]{}0,, \end{aligned}$$

which gives

$$\begin{aligned} \lim _{n\rightarrow \infty }\int _0^T(\sigma _n^+(t),G(\sigma _n^+(t)))_{p,p'}\mathrm dt&=\lim _{n\rightarrow \infty }\int _0^T(\sigma _n^+(t),G_n(\sigma _n^+(t)))_{p,p'}\,\mathrm dt\\ {}&=\int _0^T(\sigma (t),e u(t)+e\dot{u}(t))_{p,p'}\,\mathrm dt. \end{aligned}$$

Moreover, thanks to (G3) and (3.22) the sequence $\{G(\sigma _n^+)\}_n\subset L^{p'}(0,T;L^{p'}(\Omega ;{\mathbb {R}}^{d\times d}_{sym}))$ is uniformly bounded. Hence, by (3.54) and (3.67) we deriveWe combine these two facts and we obtain that for all $w\in L^p(0,T;L^p(\Omega ;{\mathbb {R}}^{d\times d}_{sym}))$

$$\begin{aligned}&0\le \lim _{n\rightarrow \infty }\int _0^T(\sigma _n^+(t)-w(t),G(\sigma _n^+(t))-G(w(t)))_{p,p'}\,\mathrm dt\\&\quad = \int _0^T(\sigma (t)-w(t),eu(t)+e\dot{u}(t)-G(w(t)))_{p,p'}\,\mathrm dt. \end{aligned}$$

In particular, we take $w:=\sigma -k b$ with $b\in L^p(0,T;L^p(\Omega ;{\mathbb {R}}^{d\times d}_{sym}))$ and $k>0$, and by dividing by *k* we get

$$\begin{aligned} 0\le \int _0^T(b(t),eu(t)+e\dot{u}(t)-G(\sigma (t)-kb(t)))_{p,p'}\,\mathrm dt. \end{aligned}$$

Since *G* is continuous, by sending $k\rightarrow 0^+$ we deduce

$$\begin{aligned} 0\le \int _0^T(b(t),eu(t)+e\dot{u}(t)-G(\sigma (t)))_{p,p'}\,\mathrm dt \end{aligned}$$

for all $b\in L^p(0,T;L^p(\Omega ;{\mathbb {R}}^{d\times d}_{sym}))$. This implies the constitutive law (2.11). $\square$

We can finally prove our main existence result Theorem 2.8.

### Proof of Theorem 2.8

It is enough to combine Lemma 3.5 with Lemmas 3.7–3.9. $\square$

We conclude this section with a uniqueness result in the space $({\mathcal {V}}\cap W^{2,2}(0,T;H))\times L^p(0,T;L^p(\Omega ;{\mathbb {R}}^{d\times d}_{sym}))$ for the weak solutions $(u,\sigma )$ to the system (2.6)–(2.8) which satisfy the initial conditions (2.9).

### Theorem 3.10

Let $(u,\sigma )\in ({\mathcal {V}}\cap W^{2,2}(0,T;H))\times L^p(0,T;L^p(\Omega ;{\mathbb {R}}^{d\times d}_{sym}))$ be a weak solution to the nonlinear viscoelastic system (2.6)–(2.8) satisfying the initial conditions (2.9). Then, the function *u* is unique. Moreover, if *G* is strictly monotone, also $\sigma$ is unique.

### Proof

Let $(u_1,\sigma _1),(u_2,\sigma _2)\in ({\mathcal {V}}\cap W^{2,2}(0,T;H))\times L^p(0,T;L^p(\Omega ;{\mathbb {R}}^{d\times d}_{sym}))$ be two weak solutions to the nonlinear viscoelastic system (2.6)–(2.8) satisfying the initial conditions (2.9).

We fix $s\in (0,T]$. If we set $u:=u_1-u_2\in {\mathcal {V}}\cap W^{2,2}(0,T;H)$, by arguing as in (3.64), we derive that *u* satisfies the following identity

3.68 $$\begin{aligned} \int _0^s(\ddot{u}(t), \varphi (t))_H\,\mathrm dt+\int _0^s(\sigma _1(t)-\sigma _2(t),e\varphi (t))_{p,p'}\,\mathrm dt=0 \end{aligned}$$

for all $\varphi \in L^{p'}(0,s;V)\cap L^2(0,s;H)$ with $\varphi (t)\in V_t^D$ for a.e. $t\in [0,s]$. Moreover, we have

3.69 $$\begin{aligned} u(0)=\dot{u}(0)=0,\qquad u(t)+\dot{u}(t)\in V^D_t\quad \text{ for } \text{ a.e. } t\in [0,T]. \end{aligned}$$

Thanks to (3.69) we can use $u+\dot{u}$ as test function in (3.68), and we get

3.70 $$\begin{aligned} \int _0^s(\ddot{u}(t),u(t)+\dot{u}(t))_H\,\mathrm dt=-\int _0^s(\sigma _1(t)-\sigma _2(t),eu(t)+e\dot{u}(t))_{p,p'}\,\mathrm dt.\nonumber \\ \end{aligned}$$

By taking into account (2.5) and (2.11), by (3.69) we have

3.71 $$\begin{aligned}{} & {} \int _0^s(\sigma _1(t)-\sigma _2(t),eu(t)+e\dot{u}(t))_{p,p'}\,\mathrm dt\nonumber \\ {}{} & {} \quad =\int _0^s(\sigma _1(t)-\sigma _2(t),G(\sigma _1(t))-G(\sigma _2(t)))_{p,p'}\,\mathrm dt\ge 0. \end{aligned}$$

Moreover, since $u\in W^{2,2}(0,T;H)$, we derive

3.72 $$\begin{aligned} \int _0^s(\ddot{u}(t),u(t)+\dot{u}(t))_H\,\mathrm dt=\frac{1}{2}\Vert \dot{u}(s)\Vert _H^2+(\dot{u}(s),u(s))_H-\int _0^s\Vert \dot{u}(t)\Vert _H^2\,\mathrm dt, \end{aligned}$$

and by Young inequality

3.73 $$\begin{aligned} |(\dot{u}(s),u(s))_H|\le \frac{1}{4}\Vert \dot{u}(s)\Vert _H^2+\Vert u(s)\Vert _H^2\le \frac{1}{4}\Vert \dot{u}(s)\Vert _H^2+T\int _0^s\Vert \dot{u}(t)\Vert _H^2\,\mathrm dt. \end{aligned}$$

Hence, by (3.70)–(3.73), for every $s\in (0,T]$ we obtain

3.74 $$\begin{aligned}{} & {} \frac{1}{4}\Vert \dot{u}(s)\Vert _H^2-(T+1)\int _0^s\Vert \dot{u}(t)\Vert _H^2\,\mathrm dt\nonumber \\{} & {} \quad \le \frac{1}{2}\Vert \dot{u}(s)\Vert _H^2+(\dot{u}(s),u(s))_H-\int _0^s\Vert \dot{u}(t)\Vert _H^2\,\mathrm dt \le 0. \end{aligned}$$

In particular, since

$$\begin{aligned}{} & {} \frac{\mathrm d}{\mathrm ds}\left( \mathrm e^{-4(T+1)s}\int _0^s\Vert \dot{u}(t)\Vert _H^2\,\mathrm dt\right) \\ {}{} & {} \quad =\mathrm e^{-4(T+1)s}\left( \Vert \dot{u}(s)\Vert _H^2-4(T+1)\int _0^s\Vert \dot{u}(t)\Vert _H^2\,\mathrm dt\right) \quad \text{ for } \text{ a.e. }\ s\in [0,T], \end{aligned}$$

thanks to (3.74) we have that the function $s\mapsto \mathrm e^{-4(T+1)s}\int _0^s\Vert \dot{u}(t)\Vert _H^2\,\mathrm dt$ is decreasing on [0, *T*], from which we deduce

$$\begin{aligned} \int _0^s\Vert \dot{u}(t)\Vert _H^2\,\mathrm dt=0\quad \text {for all}\ s\in [0,T]. \end{aligned}$$

Therefore, $\dot{u}\equiv 0$ on [0, *T*], which implies $u\equiv c$ for some constant $c\in H$. By (3.69), we have $0=u(0)=c$, that is $u_1=u_2$.

Finally, if *G* is strictly monotone, by $G(\sigma _1)-G(\sigma _2)=eu+e\dot{u}=0$, we conclude that $\sigma _1=\sigma _2$. $\square$

## Energy-dissipation balance and the viscoelastic paradox

In Theorem 2.8, we proved the existence of a solution $(u,\sigma )$ to the nonlinear viscoelastic system (2.6)–(2.8). As observed in Lemma 3.9, the displacement *u* obtained via the discretization-in-time scheme is more regular in time, more precisely $u\in W^{2,2}(0,T;H)$. This regularity allows us to prove the following energy-dissipation balance.

### Theorem 4.1

Every weak solution $(u,\sigma )\in ({\mathcal {V}}\cap W^{2,2}(0,T;H))\times L^{p}(0,T;L^p(\Omega ;{\mathbb {R}}^{d\times d}_{sym}))$ to the nonlinear viscoelastic system (2.6)–(2.8) satisfies the energy-dissipation balance

4.1 $$\begin{aligned}{} & {} \frac{1}{2}\Vert \dot{u}(s)\Vert _H^2+\int _0^s(\sigma (t),e{\dot{u}}(t))_{p,p'}\,\mathrm dt\nonumber \\{} & {} \quad = \frac{1}{2}\Vert \dot{u}(0)\Vert _H^2+\mathcal {W}(0,s;u,\sigma )\quad \text {for all} \ s\in [0,T], \end{aligned}$$

where ${\mathcal {W}}(0,s;u,\sigma )$ is the total work of $(u,\sigma )$ on the time interval $[0,s]\subseteq [0,T]$, defined as

$$\begin{aligned} {\mathcal {W}}(0,s;u,\sigma )&:=\int _0^s(f(t), \dot{u}(t)-\dot{z}(t))_H\,\mathrm dt+\int _0^s(\ddot{u}(t), {\dot{z}}(t))_H\,\mathrm dt\\ {}&\quad +\int _0^s(\sigma (t),e {\dot{z}}(t))_{p,p'}\,\mathrm dt\qquad \mathrm {for~all}\ s\in [0,T]. \end{aligned}$$

### Proof

We fix $s\in (0,T]$. By arguing as in (3.64), we derive that the pair $(u,\sigma )\in ({\mathcal {V}}\cap W^{2,2}(0,T;H))\times L^{p}(0,T;L^p(\Omega ;{\mathbb {R}}^{d\times d}_{sym}))$ satisfies

$$\begin{aligned} \int _0^s(\ddot{u}(t),\varphi (t))_H\,\mathrm dt+\int _0^s(\sigma (t),e \varphi (t))_{p,p'}\,\mathrm dt=\int _0^s(f(t),\varphi (t))_H\,\mathrm dt \end{aligned}$$

for all $\varphi \in L^{p'}(0,s;V)\cap L^2(0,s;H)$ with $\varphi (t)\in V_t^D$ for a.e. $t\in [0,s]$. Hence, if we use $\varphi :=\dot{u}-{\dot{z}}$ we obtain

$$\begin{aligned} \int _0^s(\ddot{u}(t),\dot{u}(t))_H\,\mathrm dt+\int _0^s(\sigma (t),e \dot{u}(t))_{p,p'}\,\mathrm dt={\mathcal {W}}(0,s;u,\sigma )\quad \text {for all}\ s\in [0,T]. \end{aligned}$$

Finally, since $u\in W^{2,2}(0,T;H)$, we can use the identity

$$\begin{aligned} \int _0^s(\ddot{u}(t),\dot{u}(t))_H\,\mathrm dt=\frac{1}{2}\Vert \dot{u}(s)\Vert _H^2-\frac{1}{2}\Vert \dot{u}(0)\Vert _H^2\quad \text {for all}\ s\in [0,T] \end{aligned}$$

to derive (4.1). $$\square $$

We conclude the paper by showing that in the nonlinear Kelvin–Voigt model, which is the one associated with the monotone operator

4.2 $$\begin{aligned} G(\xi ):=|\xi |^{p-2}\xi \quad \text {for}\ \xi \in {\mathbb {R}}^{d\times d}_{sym}, \end{aligned}$$

the solution to the system (2.6)–(2.8) found in Theorem 2.8 satisfies another energy-dissipation balance, which is (4.7). This implies that the crack can not propagate in time, i.e., also the nonlinear Kelvin–Voigt model of dynamic fracture exhibits the viscoelastic paradox, as discussed in the introduction.

We assume that *G* is defined by (4.2). Therefore, *G* satisfies the assumptions (G1)–(G3) and in addition it is strictly monotone. In particular, *G* is invertible and its inverse is given by

$$\begin{aligned} G^{-1}(\eta )=|\eta |^{p'-2}\eta \quad \text {for}\ \eta \in {\mathbb {R}}^{d\times d}_{sym}. \end{aligned}$$

In this case, the system (2.6) reduces to

4.3 $$\begin{aligned} \ddot{u}(t)-\mathop {\textrm{div}}\nolimits (|eu(t)+e\dot{u}(t)|^{p'-2}(eu(t)+e\dot{u}(t)))=f(t)\quad \text {in}\ \Omega \setminus \Gamma _t, t\in [0,T],\nonumber \\ \end{aligned}$$

with boundary conditions

4.4 $$\begin{aligned}&u(t)=z(t){} & {} \quad \text {on}\ \partial _D\Omega ,&\quad t\in [0,T],\end{aligned}$$

4.5 $$\begin{aligned}&|eu(t)+e\dot{u}(t)|^{p'-2}(eu(t)+e\dot{u}(t))\nu =0{} & {} \quad \text {on}\ \partial _N\Omega \cup \Gamma _t,&\quad t\in [0,T], \end{aligned}$$

and initial conditions

4.6 $$\begin{aligned}&u(0)=u^0,\quad {\dot{u}}(0)=u^1 \quad \text {in}\ \Omega \setminus \Gamma _0. \end{aligned}$$

According to Definition 2.3, we say that $u\in {\mathcal {V}}$ is a *weak solution* to the nonlinear Kelvin–Voigt system (4.3)–(4.5) if $u(t)-z(t)\in V_t^D$ for all $t\in [0,T]$ and the following identity holds:

$$\begin{aligned}{} & {} -\int _0^T(\dot{u}(t),{{\dot{\varphi }}}(t))_H\,\mathrm dt+\int _0^T(|eu(t)+e\dot{u}(t)|^{p'-2}(eu(t)+e\dot{u}(t)),e \varphi (t))_{p,p'}\,\mathrm dt\\{} & {} \quad =\int _0^T( f(t),\varphi (t))_H\,\mathrm dt \end{aligned}$$

for all $\varphi \in {\mathcal {D}}$. By Theorems 2.8 and 3.10 we know that there exists a unique weak solution $u\in {\mathcal {V}}\cap W^{2,2}(0,T;H)$ to (4.3)–(4.5) which satisfies the initial conditions (4.6). Moreover, by Theorem 4.1 the function *u* satisfies the identity (4.1).

We want to show that the energy-dissipation balance (4.1) can be rephrased just in terms of *u*. Given $u\in {\mathcal {V}}\cap W^{2,2}(0,T;H)$, we define the mechanical energy ${\mathscr {E}}$ at time $s\in [0,T]$ as

$$\begin{aligned} {\mathscr {E}}(s;u):=\frac{1}{2}\Vert \dot{u}(s)\Vert _H^2+\frac{1}{p'}\Vert eu(s)\Vert _{p'}^{p'}, \end{aligned}$$

the energy dissipated by the viscous term ${\mathscr {V}}$ on the time interval $[0,s]\subseteq [0,T]$ as

$$\begin{aligned} {\mathscr {V}}(0,s;u):=\int _0^s(|eu(t)+e\dot{u}(t)|^{p'-2}(eu(t)+e\dot{u}(t))-|eu(t)|^{p'-2}eu(t),e\dot{u}(t))_{p,p'}\,\mathrm dt, \end{aligned}$$

and the total work ${\mathscr {W}}$ on the time interval $[0,s]\subseteq [0,T]$ as

$$\begin{aligned} {\mathscr {W}}(0,s;u):=&\int _0^s(f(t), \dot{u}(t)-{\dot{z}}(t))_H\,\mathrm dt+\int _0^s(\ddot{u}(t), {\dot{z}}(t))_H\,\mathrm dt \\&+\int _0^s(|eu(t)+e\dot{u}(t)|^{p'-2}(eu(t)+e\dot{u}(t)),e \dot{z}(t))_{p,p'}\,\mathrm dt. \end{aligned}$$

### Remark 4.2

For $p=2$, we have

$$\begin{aligned} {\mathscr {V}}(0,s;u)=\int _0^s\Vert e\dot{u}(t)\Vert ^2_H\, \mathrm dt, \end{aligned}$$

which corresponds to the viscous dissipation term in the linear Kelvin–Voigt model. Moreover, since $G^{-1}$ satisfies (G1), we deduce that

$$\begin{aligned} (G^{-1}(\eta _1)-G^{-1}(\eta _2))\cdot (\eta _1-\eta _2)\ge 0\quad \text {for all}\ \eta _1,\eta _2\in {\mathbb {R}}^{d\times d}_{sym}, \end{aligned}$$

and by choosing $\eta _1=eu(t)+e{\dot{u}}(t)$ and $\eta _2=eu(t)$ we derive

$$\begin{aligned} {\mathscr {V}}(0,s;u)\ge 0\quad \text {for every}\ s\in [0,T]. \end{aligned}$$

Therefore, ${\mathcal {V}}$ can be seen as the analogous of the viscous dissipation term in the nonlinear setting.

Thanks to Theorem 4.1 and (4.2), we derive the following result.

### Corollary 4.3

Every weak solution $u\in {\mathcal {V}}\cap W^{2,2}(0,T;H)$ to the nonlinear Kelvin–Voigt system (4.3)–(4.5) satisfies the energy-dissipation balance

4.7 $$\begin{aligned} {\mathscr {E}}(s;u)+{\mathscr {V}}(0,s;u)= {\mathscr {E}}(0;u)+{\mathscr {W}}(0,s;u)\quad \text {for all}\ s\in [0,T]. \end{aligned}$$

### Proof

By Theorem 4.1, we know that *u* satisfies the energy dissipation balance (4.1). Moreover, for the nonlinear operator *G* given by (4.2) we observe that

$$\begin{aligned}&\int _0^s(\sigma (t),e\dot{u}(t))_{p,p'}\,\mathrm dt\\&\quad =\int _0^s(|eu(t)+e\dot{u}(t)|^{p'-2}(eu(t)+e\dot{u}(t)),e\dot{u}(t))_{p,p'}\,\mathrm dt\\&\quad =\int _0^s(|eu(t)+e\dot{u}(t)|^{p'-2}(eu(t)+e\dot{u}(t))-|eu(t)|^{p'-2}eu(t),e\dot{u}(t))_{p,p'}\,\mathrm dt\\&\qquad +\int _0^s(|eu(t)|^{p'-2}eu(t),e\dot{u}(t))_{p,p'}\,\mathrm dt\\&\quad =\int _0^s(|eu(t)+e\dot{u}(t)|^{p'-2}(eu(t)+e\dot{u}(t))-|eu(t)|^{p'-2}eu(t),e\dot{u}(t))_{p,p'}\,\mathrm dt\\&\qquad +\frac{1}{p'}\Vert eu(s)\Vert _{p'}^{p'}-\frac{1}{p'}\Vert eu(0)\Vert _{p'}^{p'}. \end{aligned}$$

Indeed, $u\in W^{1,p'}(0,T;V)$, which implies that the map $t\mapsto \Vert eu(t)\Vert _{p'}^{p'}$ is absolutely continuous on [0, *T*] with

$$\begin{aligned} \frac{\mathrm d}{\mathrm dt}\Vert e u(t)\Vert _{p'}^{p'}=p'(|eu(t)|^{p'-2}eu(t),e\dot{u}(t))_{p,p'}\quad \text {for a.e.}\ t\in [0,T]. \end{aligned}$$

By combining the previous identity with (4.1) we derive (4.7). $\square$

As a consequence of Corollary 4.3, we deduce that for every weak solution $u\in {\mathcal {V}}\cap W^{2,2}(0,T;V)$ to the nonlinear Kelvin–Voigt system (4.3)–(4.5) the crack can not grow in time. Indeed, as explained in the introduction, according to the Griffith criterion there is a balance between the mechanical energy dissipated and the energy used to increase the crack. In the nonlinear Kelvin–Voigt model (4.3)–(4.5), this reads as

$$\begin{aligned} {\mathscr {E}}(s;u)+{\mathcal {H}}^{d-1}(\Gamma _t\setminus \Gamma _0)+{\mathscr {V}}(0,s;u)= {\mathscr {E}}(0;u)+{\mathscr {W}}(0,s;u)\quad \text {for all}\ s\in [0,T]. \end{aligned}$$

Since the energy dissipated by the crack growth, which is ${\mathcal {H}}^{d-1}(\Gamma _t\setminus \Gamma _0)$, does not appear in (4.7), we derive that for the weak solution $u\in {\mathcal {V}}\cap W^{2,2}(0,T;H)$ to (4.3)–(4.5) given by Theorem 2.8 we must have ${\mathcal {H}}^{d-1}(\Gamma _t\setminus \Gamma _0)=0$ for every $t\in [0,T]$. Hence, the crack associated with *u* does not increase in time. We point out that this phenomenon, called viscoelastic paradox, is the same which arises in linear Kelvin–Voigt models, as shown in [10, 26].
